# High aspect-ratio quadrilateral flexible silica optical fibers for enhanced multi-parameter sensing

**DOI:** 10.1038/s41467-026-72416-6

**Published:** 2026-04-29

**Authors:** Pawel Maniewski, Robin Hartley, Timothy Lee, Matthew Whitaker, Bruno Moog, Martynas Beresna, Janice M. Dulieu-Barton, Christopher Holmes

**Affiliations:** 1 Optoelectronic Research Centre, University of Southampton, Southampton, UK; 2 Department of Applied Physics, KTH Royal Institute of Technology, Stockholm, Sweden; 3 Bristol Composites Institute, University of Bristol, Bristol, UK; 4 School of Engineering, University of Southampton, Southampton, UK

**Keywords:** Fibre optics and optical communications, Optical sensors, Mechanical engineering, Sensors and biosensors

## Abstract

A planar silica optical fiber platform, characterized by an ultra-high aspect ratio, is introduced, termed high aspect ratio flat fiber (HARFF). Non‑circular fibers offer unique opportunities for sensing, yet their mechanical behavior and fabrication have remained challenging. The HARFF is a specialty optical fiber with a quadrilateral cross-section, which is demonstrated to provide enhanced mechanical compliance and tunable microstructures that enable highly sensitive multiparameter sensing. Using laser-based processing combined with stack-and-draw fabrication, HARFFs are engineered with tailored mechanical properties and microstructure. By exploiting their flat geometry, we demonstrate pressure sensors with sensitivities up to three orders of magnitude higher than those based on conventional circular fibers. Furthermore, using a multi-material microstructure, enhanced temperature sensitivity was demonstrated using a Sn-alloy-filled HARFF design. These findings highlight the potential of HARFFs, opening new avenues for innovation in non-circular high aspect-ratio optical fiber geometries.

## Abstract (web summary)

This work introduces thin, mechanically compliant high-aspect-ratio silica fibers that enable enhanced sensitivity to external stimuli, outperforming conventional optical fibers and opening new possibilities for advanced monitoring technologies.

## Introduction

Advances in silica optical fibers have consistently driven transformative breakthroughs, reshaping industries and everyday life. By introducing novel designs, optical fibers continue to push boundaries, set new standards, and overcome longstanding limitations in signal delivery, speed, and efficiency^1–4^. In particular, optical fiber-based sensors benefit greatly from advances made in optical fiber fabrication techniques, achieving enhanced precision, resilience, and versatility across a wide range of applications, from aerospace to biomedical diagnostics and beyond. Artificial Intelligence and digitalization also benefit from advances in optical fiber sensing, where robust, high-resolution, and high-fidelity information permits new, enhanced predictive maintenance and adaptive control^5–8^.

The mechanical robustness of silica optical fiber stems from the strong, covalent Si-O bonds, making them particularly suited to chemically aggressive, high-temperature, and high-pressure environments^9–11^. Optical fibers are also offering integration advantages over traditional electronic sensors through their compact size, multiplexing capability, and inherent immunity of the signal to electromagnetic interference. Silica optical fibers provide a flexible platform, where pliability is inherently linked to the small fiber diameter. This dimensional effect simplifies fiber-to-structure integration while maintaining optical integrity through the minimization of microbend losses through sufficient stiffness. More broadly, embedding multiple sensing elements along a single compact optical fiber reduces cabling and routing complexity faced by electronic alternatives, simplifying integration into engineered structures. Optical fiber further offers scalability through fiber drawing, permitting cost-effective production that can subsequently be easily deployed across diverse applications^12–16^.

Many traditional fabrication approaches limit the design space of optical fiber preform to an axisymmetric layout of a centrally located core and cladding^16^. For more complex specialty optical fibers, manual stacking or physical machining of the initially solid cross-section preform is often used, e.g., to create microstructure^17–20^. In these instances, however, the design space is still often limited to the circular cross-section of the fiber’s cladding. Furthermore, the most common optical fiber with a core-cladding solid structure is only effective in measuring strains produced by forces aligned with the fiber axis, while being limited when monitoring transverse loads, including those induced upon hydrostatic loading. The current hydrostatic sensitivity of Fiber Bragg Gratings (FBGs) in standard single-mode silica optical fiber is 0.65 pm/MPa^21^. This limits the usability of optical fiber in applications where low-profile transverse load monitoring is desired^22^, in high-value assets such as composite structures. Incorporating microstructure, i.e., air channels running parallel to the waveguide, can significantly enhance this sensitivity^23^. Nonetheless, even for a microstructured fiber, inherent hydrostatic pressure resolution is of the order 0.23 MPa^24,25^, assuming the use of an FBG and a typical commercial interrogator. Although higher resolutions might be obtained by coupling fiber to a mechanical structure, this is not the same as inherent pressure sensitivity, as it comes at the compromise of form-factor.

Flat fibers, characterized by a nominally rectangular cross-section, have emerged as a promising platform for new sensing capability, including radiation dosimetry^26^, spectral interrogation^27^, and multiparameter sensing^28^. Traditionally, flat fibers have been fabricated through physical machining of round preforms or a collapsed draw process^26,28–30^. Machined preforms allow for reduced fiber thickness in comparison to circular counterparts, yet their width remains constrained by the preform diameter, resulting typically in a relatively low aspect ratio. To achieve higher aspect ratios, up to 10:1, collapsed drawing has been employed, where vacuum-assisted fiber drawing induces a flat cross-section^26,30^. The inherent axial symmetry of this process leads, however, to multiple local energy minima during collapse, making the final geometry sensitive to perturbations. As a result, the collapse often initiates non-uniformly, introducing twist and asymmetry, leading to poor control over the final shape and microstructure. Stack-and-draw of flat fiber has also been demonstrated as an alternative approach^31^, but thus far has been limited to soda-lime glass, which exhibits poor environmental durability and suboptimal optical performance relative to fused silica. Prior work has also been constrained to unitary (1:1) aspect ratios when incorporating microstructure. In this context, fused silica offers markedly superior chemical resistance and optical clarity; its high viscosity also demands prolonged high-temperature processing for effective silica-to-silica bonding, conditions that can also cause warpage of the preform during its fabrication and necessitate complex production strategies.

The high aspect ratio flat fiber (HARFF), described in the present paper, is a new type of optical fiber platform that utilizes the optical performance and environmental resilience of fused silica with the mechanical adaptability and scalability of optical fiber fabrication. Importantly, HARFFs feature a high-aspect-ratio ribbon-like cross-section, enabling unidirectional flexibility. By leveraging fiber-drawing, HARFFs can be produced in over a hundred meter-scale lengths with micrometer thickness and millimeter width. The combination of geometry and fabrication strategy opens new opportunities for applications ranging from conformal biosensing and flexible interconnects to large-area structural health monitoring, where robustness, integration versatility, and environmental resilience are critical.

The scope of the work described in the following sections of the paper is to underline the development and potential applications of high-aspect-ratio microstructure. HARFFs are fully compatible with commercial fibers, enabling seamless and straightforward integration into existing optical systems and infrastructure. A key feature of the HARFF is the ability to integrate a relatively large microstructure in the fiber to enhance and tailor the fiber sensitivity to the sensor application. By adapting stack-and-draw with an additional step of laser-postprocessing of the preform, chemically doped, low-loss waveguiding features in strategically desired positions were integrated in the HARFF platform to leverage enhanced sensitivity linked to a large microstructure.

**Fig. 1:**
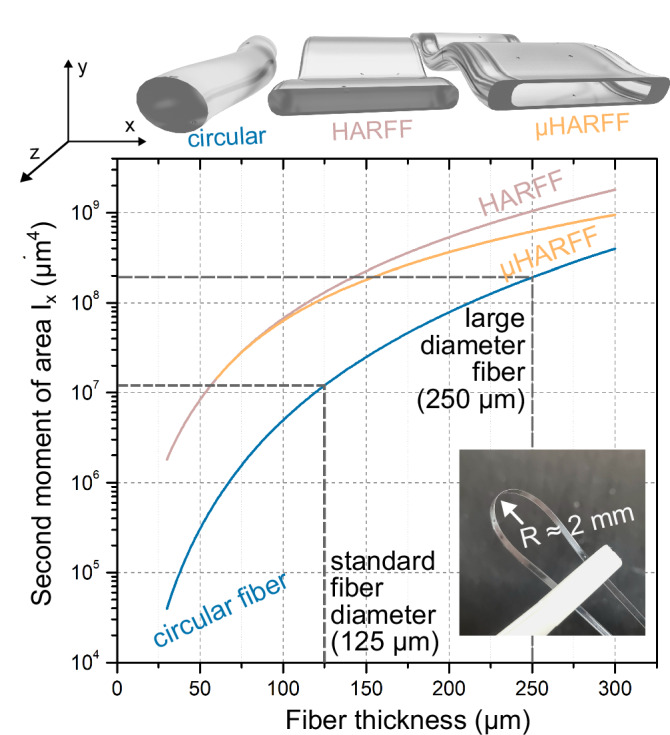
Comparison of the second moment of area (I_x_) between a circular fiber and 800 µm wide High Aspect-Ratio Flat Fiber (HARFF). In the case of microstructure HARFF (µHARFF), the wall thickness of 30 µm was used. The inset shows a photograph demonstrating the minimal bend radius of an uncoated HARFF having a cross-section of 850 µm by 40 µm.

The first mechanical advantage of HARFFs over conventional axisymmetric fibers lies in their flexural rigidity, and the second is the ability to integrate high-aspect ratio microstructure, e.g., air-filled elliptical channels that can alter the optomechanical performance of the fiber. These initially air-filled channels can also be filled with other, dissimilar materials to further tune performance. Figure 1 shows the second moment of area about the lateral axis (*I*_*x*_) for conventional optical fibre, compared to solid-core HARFF, and single channel microstructured HARFF (µHARFF), with a width of 800 μm (see Eq. 1).

1 $${I}_{x}={\iint }_{R}\,{y}^{2}\,{dx}\,{dy}$$

Notably, HARFF with a thickness of approximately 55 µm exhibits comparable lateral flexural rigidity to standard 125 μm diameter optical fibers. For thicker HARFFs, i.e., > 100 µm, *I*_*x*_ is comparable to the common large diameter round fibers 250 µm. This ensures that thicker HARFF are both rigid, while being flexible enough to be wound. The latter is pivotal in terms of scalability for any specialty optical fiber. Lastly, the vertical second moment of area (*I*_*y*_) can be tailored independently by adjusting the width for any given desired thickness, an approach not feasible for an axisymmetric fiber. Moreover, the rectangular cross-section of HARFF mitigates buckling and twisting common in the case of circular fibers.

The second mechanical advantage of the µHARFF platform, linked to its large cross-sectional width, is the ability to integrate microstructural features that enhance sensitivity to external stimuli^24^. Under mechanical loading, the microstructure geometry generates an anisotropic strain field within the cross-section, inducing birefringence in the optical waveguide. This strain-induced birefringence modulates the response of FBGs written in the waveguide and forms the basis of the sensing mechanism. Strain-induced birefringence (*ΔB*) is governed by the difference between the two transverse principal strains. By convention, $B\equiv {n}_{{slow}}-{n}_{{fast}} > 0$, so the slow axis is aligned with the direction of maximum transverse principal strain ${\varepsilon }_{1}$ and the fast axis with the minimum ${\varepsilon }_{2}$. Under the assumption of small intrinsic birefringence, the strain-induced birefringence may be written as

2 $$\varDelta B\approx -\frac{{{n}_{{eff}}}^{3}}{2}\left({p}_{11}-{p}_{12}\right)\left({\varepsilon }_{1}-{\varepsilon }_{2}\right)$$

where ${n}_{{eff}}$ is the mean effective refractive index of the two orthogonal polarized guided modes and the coefficients ${p}_{11}$ and ${p}_{12}$ are the strain-optic coefficients of fused silica, taken as 0.121 and 0.270, respectively.

In the µHARFF platform, tailoring the cross-section geometry, through size, shape, and spacing of air channels, provides a direct means to control the magnitude of (*ε*_1_–*ε*_2_)$\left({\varepsilon }_{1}-{\varepsilon }_{2}\right)$ at the waveguide, and hence the birefringence sensitivity^32^. In the present study, hydrostatic pressure is considered as a representative external stimulus. Accordingly, a finite element method (FEM) model was developed to evaluate the strain and birefringence of candidate µHARFF geometries under hydrostatic loading and to identify designs that maximize pressure sensitivity through a geometric parameter sweep. It should be noted that the model is intended to be used as a comparative tool to assess the effect of varying µHARFF geometries on the *ΔB* values at the waveguide position. To provide quantitative values of *ΔB*, a knowledge of localized residual stress arising from the femtosecond laser fabrication of the sensor and the optical fibre draw would be required, as well as an accurate representation of the geometry, including dimensional tolerances and edge and corner radii.

**Fig. 2:**
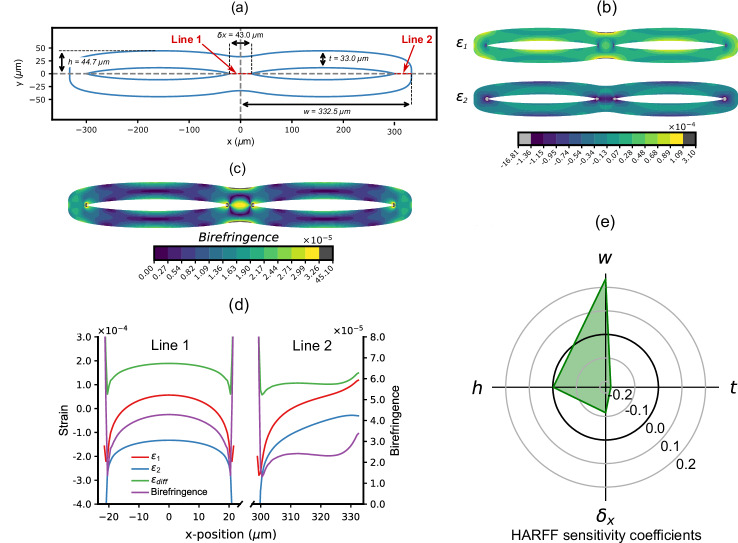
FEM analysis of the twin-channel µHARFF cross-section. **a** FEM geometry corresponding to the twin-channel µHARFF with Lines 1 and 2 indicating extraction paths. **b** Principal strain fields ${\varepsilon }_{1}$ and ${\varepsilon }_{2}$; **c** Birefringence distribution derived from ${\varepsilon }_{{diff}}$. (d) Principal strains (${\varepsilon }_{1}$, ${\varepsilon }_{2}$), strain differences $({\varepsilon }_{{diff}})$, and birefringence plotted along Lines 1 and 2. Spider plot (**e**) showing the sensitivity of birefringence at (*x* = 0, *y* = 0) to changes in the µHARFF geometric parameters.

The FEM study (see Methods) considered a representative µHARFF incorporating a twin-channel cross-sectional microstructure (see Fig. 2a). Conceptually, the cross-section maximizes strain-induced birefringence at the potential waveguide location ($x=0,{y}=0$) by increasing the difference between the transverse principal strains, ${\varepsilon }_{{diff}}={\varepsilon }_{1}-{\varepsilon }_{2}$. This is achieved by promoting asymmetric deformation of the cross-section under external pressure, arising from the increased compliance of the structure in the $y$-direction. Figure 2b shows the spatial distributions of the transverse principal strains ${\varepsilon }_{1}$ and ${\varepsilon }_{2}$ under external hydrostatic loading, obtained by transformation of the Cartesian strain tensor at the integration point of each element. Finally, the spatial distribution of the local strain-induced birefringence (Eq. 2) is shown in Fig. 2c. Figure 2d shows the variation of ${\varepsilon }_{1}$, ${\varepsilon }_{2}$, ${\varepsilon }_{{diff}}$ and $\varDelta B$ along the $x$-direction at $y=0$ (Lines 1 and 2 in Fig. 2a). Although the largest $\varDelta B$ occurs near the tips of the microstructure, these regions are characterized by high strain gradients and proximity to the fiber surface, making them unsuitable for waveguide placement due to fabrication constraints and evanescent field losses. In contrast, the central region $x=0$ provides a broad zone of elevated $\varDelta B$ at a sufficient distance from the fiber surfaces.

Lastly, a parametric sensitivity study was conducted to assess how variations in the µHARFF cross-section influence strain-induced birefringence at the waveguide location. The geometric parameters $w$, $h$, $t$ and $\delta x$ were varied over prescribed ranges about the baseline geometry (Fig. 2a). To quantify the relative influence of each parameter on the waveguide birefringence, a linear regression model was fitted to the normalized inputs and output, yielding sensitivity coefficients for each geometric variable. The resulting coefficients are summarized in the spider plot in Fig. 2e, which highlights the dominant trends. *ΔB* is most sensitive to reductions in wall thickness ($t$) and increases in cross-section width ($w$), indicating that higher aspect ratio designs provide enhanced sensitivity. These results provide quantitative guidance for the design of future µHARFF geometries.

In this work, to empirically demonstrate the functionality of the HARFF, a proof-of-concept study was conducted that utilizes a laser-written Bragg grating-based Fabry-Perot cavity (FP-FBG) design to obtain enhanced inherent sensitivity. For instance, leveraging 80 µm thick and 655 µm wide microstructured HARFF as a pressure sensor, inherent sensitivity resolution in the order of single kPa was demonstrated using standard optical interrogation, thereby representing an improvement of up to three orders of magnitude over the earlier mentioned similar sensors constructed in commercial silica fibers. Here, enhancement refers to an increased sensitivity linked to pressure-induced birefringence response upon hydrostatic loading, rather than an enhancement of Bragg wavelength shift associated with axial strain in conventional FBG sensors^32^. Moreover, a peak-to-valley (PV) sensitivity of 7.24 dB/MPa is obtained with the HARFF. Additionally, by creating a hybridized, multi-material, alloy-filled microstructure HARFF, an enhanced temperature sensor is demonstrated that provides resolutions of up to 12 pm/K and 0.075 dB/K. It is demonstrated that these advances open new design potential for specialty optical fiber to address emerging needs for high-performance sensing and future innovations in non-circular fiber optics.

## Results

### Fabrication of high aspect-ratio flat fibers

Similarly to the standard optical fiber fabrication, HARFF manufacturing can be divided into two main steps: preform preparation and fiber drawing. The preform manufacturing leverages a custom, laser-based glass processing system, which mitigates several limitations observed with traditional glass-fabrication or stack-and-draw. Advantages include high confinement of the laser beam and thus controlled thermal exposure, which was observed to mitigate surface-tension-related deformation of the preform, associated with traditional flame-based processing methods. The laser-based system that enabled cutting, glass deposition, and welding of silica-based components to form the HARFF preform has been used in prior work for laser-based manufacture of circular optical fiber, which is described in detail in refs. ^33,34^.

**Fig. 3:**
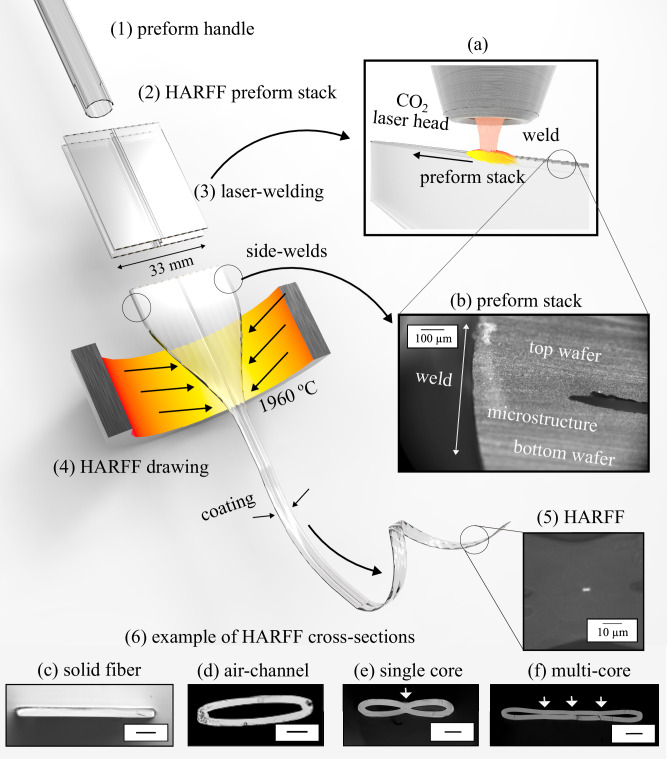
Schematic of fabrication of High Aspect-Ratio Flat Fibers (HARFFs). (1-2-3) preform stacking and laser-welding ((**a**) laser-weld used to seal the sides of the stack); micrograph (**b**) shows a typical cross-section of the weld with visible chipping in the diced-through microstructure (4) fiber drawing in traditional conductive optical fiber draw tower, and (5) typical micrograph of the core region (bottom-illuminated) and example cross-sections of **c** solid and **d**–**f** µHARFFs, where waveguides are marked with arrows. The scale bars correspond to 80 µm.

The main steps involved in the fabrication of HARFF preforms are depicted in Fig. 3. First, 1 mm thick flat silica-wafers and a germanium-doped fused silica core rods (*n*_*core*_ ≈ 1.4749, at *λ* = 1300 nm) were laser-cut to the desired dimensions, using a focused CO_2_ laser (λ = 10.6 µm). Next, the preform stack was assembled and laser-welded along the edges to create permanent and robust bonds between each component in the stack. This selective welding allowed the definition of an air-channel geometry in the HARFF, essential in the manufacturing process of µHARFF. To create the joint between the components in the stack, a defocused CO_2_ laser spot size of approximately 2 mm and a laser power of up to 60 W was used. Upon exposure to the laser beam, the energy was absorbed by the components, causing them to soften locally, resulting in a localized *melt pool*. Here, the reduced viscosity and surface tension of the softened glass allowed the reflow of the material filling gaps between the components. As the material cooled and solidified, a durable and airtight weld was formed between the components (see Fig. 3a, b). Given a relatively high viscosity of softened silica, welding was performed at a speed of 0.5 mm/s. The air-tight glass-to-glass welds allowed the microstructure to be pressurized during fiber drawing, so that the shape and fiber thickness can be controlled; examples are shown in Fig. 3d–f. Lastly, a circular silica-tube (OD of 10 mm) was laser-welded to the top of the preform, so as provide a mounting handle for a standard chuck of the fiber draw tower. In the case of the preforms used in this work, the tube was connected to a pressure-feedback system so that constant atmospheric pressure could be maintained in the microstructure during the drawing process.

The preform was drawn into HARFF using a conventional draw tower, equipped with a conductive furnace with an aperture of 35 mm. The furnace was used to heat the lower end of the preform to a drawing temperature of approximately 1960 °C. Subsequently to the initial drip and upon reaching equilibrium in the neckdown, the draw parameters remained unchanged. Upon drawing, the preform was fed into the furnace at a speed of 1 mm/min, while the capstan speed was ramped up to 1.5 m/min. Before collecting the fiber onto the capstan, a UV-curable protective coating (ORMOCER CBS-106) was applied to the fiber via atomizers and cured, protecting the glass from surface flaws and handling-induced micro-defects, which are the primary origin of strength degradation in uncoated silica. Using the abovementioned method, over 120 m of stable continuous HARFF was drawn from a single preform. This length was limited by the relatively short, 150 mm, length of the proof-of-concept preforms. To assess uniformity, the drawn, 12 m long test piece HARFF shown in Fig. 3e, was cleaved at 1 m intervals. The fiber showed up to ±2.3% width, ±6% thickness. Six months after drawing, the fiber continued to withstand proof-test loads exceeding 30 N.

**Fig. 4:**
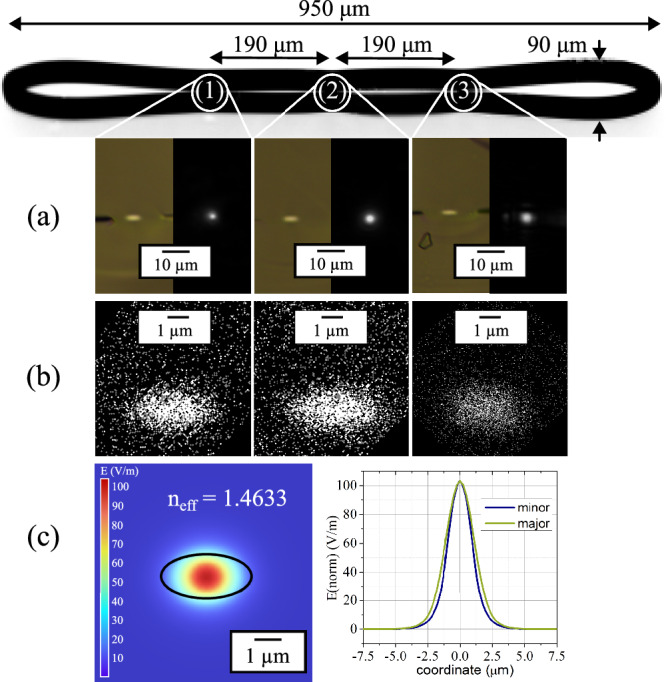
Typical cross-section of multi-core µHARFF (AR ≈ 10:1) with three parallel waveguides (labeled (1), (2), and (3)) separated by microstructure. **a** micrographs and near field mode patterns (λ = 1550 nm), **b** EDX Ge-doping maps, and **c** simulation of neff and simulated fundamental mode pattern (λ = 1550 nm) based on the dopant distribution for each waveguide.

The fibers were cleaved, and the obtained cross-sections were initially examined using optical microscopy, as shown in Fig. 3c–f. In Fig. 4a, near-field mode pattern (at *λ* = 1550 nm) is shown. Numerical aperture (NA) was relatively consistent across cores and estimated to be approximately 0.13 ± 0.02, respectively, based on a Gaussian fit of a near-field mode patterns evolution recorded for each core, at 25 known distances from the cleaved end-face (*λ* = 1550 nm). The obtained Ge-doping region was evaluated additionally via Energy Dispersion Spectroscopy (EDX). The originally round Ge-doped core rod was visibly deformed during the drawing process, resulting in an elliptical region where Ge distribution was clearly identifiable via EDX, shown in Fig. 4b. The core shape was thus approximated as an ellipse with major and minor axes of 4.7 µm and 2.3 µm, respectively. Using this geometry and the doping profile of the initial rod, the simulated fundamental mode pattern at *λ* = 1550 nm and its corresponding effective refractive index (*n*_*eff*_), obtained via COMSOL, are presented in Fig. 4c. In the case of single- and multi-core µHARFF, a transmission loss of approximately 0.16 dB/m at *λ* = 1550 nm was estimated using the cut-back technique, using initially 15 m long µHARFF section, and 5–7 consecutive cutbacks. The optical time-domain reflectometer (OTDR) did not reveal core discontinuities or noticeable scattering centers throughout the wound fiber. Due to the evident ellipticity of the waveguides, with a minor-semiaxis of up to 1.1 µm (see Fig. 3), a degree of bend sensitivity was expected, especially for longer wavelengths, which was also pronounced by a slight asymmetry in fundamental mode intensity distribution between major and minor axes (see Fig. 4c). It is noteworthy that the germanium doped fused silica core rods used to manufacture the HARFFs do not produce color centers or absorption bands that require specific evaluation.

**Fig. 5:**
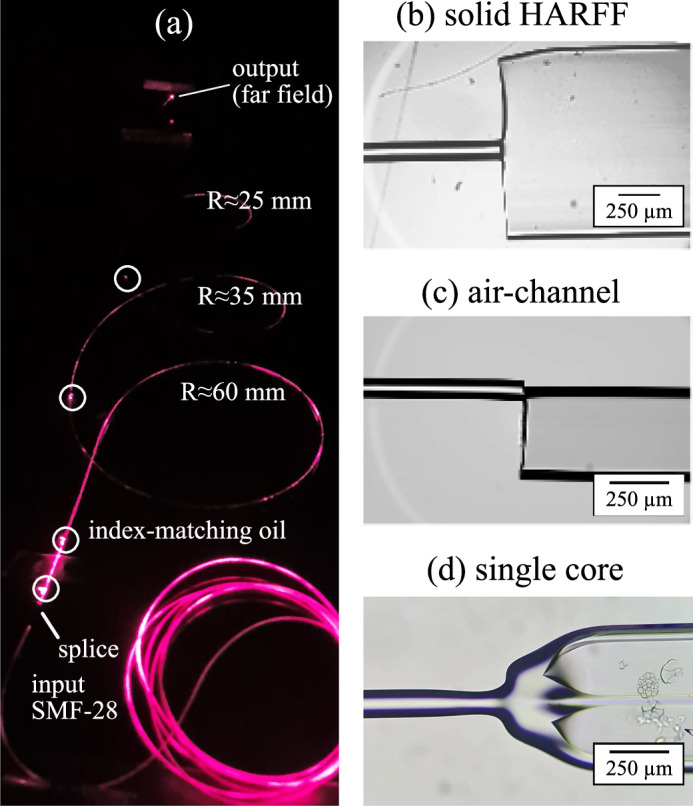
Splicing of various HARFFs with commercial fibers. photograph (**a**) of ca. 1.5 m uncoated, single-core µHARFF spliced with SMF-28e+ fiber, guiding coupled red-diode light. The µHARFF was wound and twisted in three consecutive loops; twists and bends, along with index-matching oil (*n* ≈ 1.47), were used to remove cladding modes and demonstrate stable waveguiding. Micrographs of splice regions between commercial single-mode fiber (125 µm), and HARFFs: **b** solid, **c** sidewall of a single air-channel µHARFF, **d** single-core µHARFF.

To demonstrate functional HARFF-based devices that can be integrated with standard components, various µHARFFs were cleaved using a commercial cleaver equipped with a diamond blade and spliced with a commercial single-mode fiber. The parameters were experimentally optimized and demonstrated repeatability and robustness of the splicing approach, providing a consistent geometry at the junction between the HARFFs and circular fiber. The specific splicing parameters were tuned depending upon HARFF geometry and specific application, as shown in Fig. 5. For example, in the case of the twin-channel µHARFF, it was desirable to collapse the microstructure in the splice region to create an air-tight splice, required for hydrostatic pressure sensing (as shown in Fig. 5d). Therefore, to seal the microstructure, a CO_2_ laser pulse (*t* = 1 s) was used to melt the end of the fiber and allow material to reflow to close the channels. In the case of the single air-channel µHARFF (Fig. 5c), the collapse and side-wall deformation were not desired; therefore, a shorter pulse (0.125 s) was used. The measured splice loss, typically <3 dB, was highly dependent on HARFF cleave quality and modal-mismatch. While acceptable performance was obtained, further reduction in splice loss can be achieved by tailoring HARFF core dimensions to better match the mode field diameter of standard fibers and by minimizing modal mismatch during fusion. These straightforward optimizations, combined with precise cleaving and alignment, would enable robust integration of HARFF into existing fiber systems without requiring fundamentally new splicing techniques.

### Proof of concept: sensitivity of the High Aspect-Ratio Flat Fibers

To highlight unique features of HARFF, and as proof-of-concept, simple µHARFF-based sensors were produced to demonstrate enhancements in both *(i)* pressure and *(ii)* temperature sensing. For the pressure sensor, the capability to measure changes in pressure is dependent on how the pressure load is transmitted through the optical fiber to the waveguide, which in turn is dependent on the shape of the µHARFF microstructure. According to Eq. 2, the sensitivity is directly related to the birefringence in the µHARFF microstructure generated by the photoelastic effect. Next to hydrostatic pressure, in the case of enhanced temperature measurement, to increase sensitivity and maximize birefringence response, the single-channel µHARFF was filled, post-draw, with Sn-based alloy (*T*_*melting*_ ≈ 220 °C). Here, it is more straightforward to visualize how the enhanced sensor response is generated. The functionality is based on the mismatch of the coefficients of thermal expansion (CTE) of the silica glass and the metal alloy (approximately $0.5\,\times \,{10}^{-6}\,{{{{\rm{K}}}}}^{-1}$ for the glass and $23.0\,\times \,{10}^{-6}\,{{{{\rm{K}}}}}^{-1}$ for an Sn-alloy). Conceptually, the metal alloy has a much greater CTE and will be constrained by the glass. The resulting misfit will generate both radial and tangential stresses in the glass and create a change in birefringence that is proportional to any temperature change. The induced stress modulates the birefringence in the glass, enabling a thermally tunable sensor. The thermal expansion will be greatest across the width of the µHARFF, hence the optimal position of the waveguide is evident.

To infer changes in birefringence, the HARFF-based sensors contain two spatially separated but identical period fiber Bragg gratings (FBGs). As these gratings were of identical period, a Fabry-Pérot cavity (FP-FBG) was formed between them. When unpolarized light was launched into the cavity, the summation of the generated spectra can be used to infer changes of birefringence, by means of a Vernier effect^35^. Here, the pressure-induced strain of the µHARFF alters the birefringence in the waveguide to affect the effective modal index of orthogonal polarization states, altering the contrast of the Fabry-Pérot fringe, i.e., the peak-to-valley (PV) power levels of the resulting spectra thus varied with external load, i.e., as pressure or temperature is changed.

**Fig. 6:**
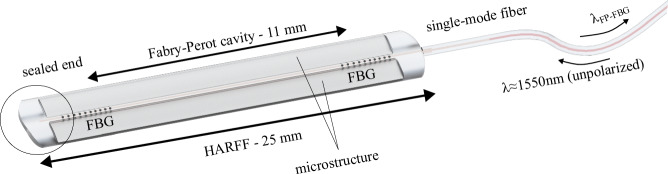
Schematic of a µHARFF-based sensor. The sensor was laser-sealed at either end and contained a FP-FBG cavity (not to scale).

An FP-FBG cavity (see Fig. 6) was written at the center of a 25 mm length of the µHARFF (see Fig. 3e), utilizing a pair of FBGs inscribed in a waveguide, which was spliced to SMF-28e + , using the splicing approach described in *Methods*. Subsequently, two FBGs were inscribed in the waveguide using the point-by-point method^36^. Each FBG was 3 mm long with an 11 mm separation between its midpoints. The cavity length corresponds to a ~80 pm FSR, which ensured visibility of spectral fringes on the spectrum analyzer, having 20 pm resolution, used for in-situ characterization during fabrication. The formed cavity was longitudinally centered in µHARFF, set away from the CO_2_ laser-sealed ends.

**Fig. 7:**
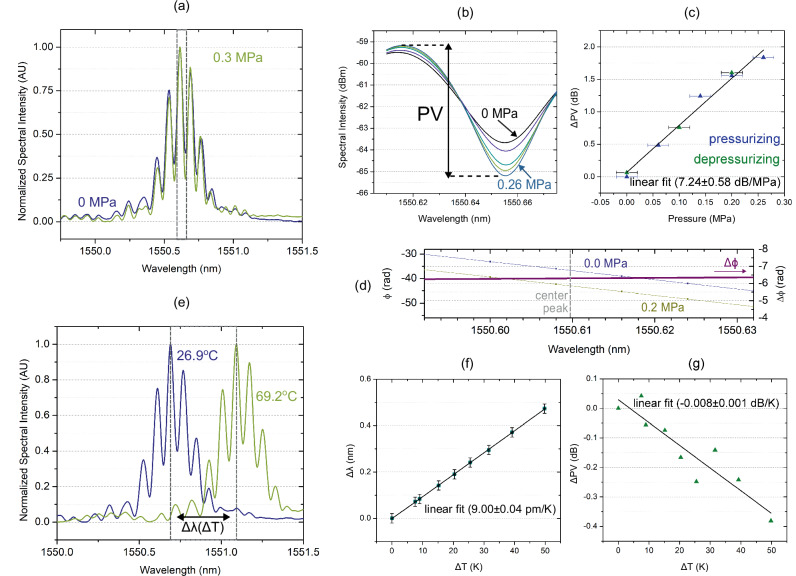
Performance of the proof-of-concept pressure sensor. Spectral response of **a** µHARFF-based pressure sensor with visible fringes, (**b**) typical PV change for different pressures (see the dashed fringe in (**a**)), and (**c**) linear fit of pressure sensitivity during pressurizing (in blue) and depressurizing the vessel (in green), and **d** phase response, ϕ, with the differential phase, Δϕ, of the sensor under target stimulus. Spectral shift (**e**) of the center fringe in elevated temperatures, and (**f**) it corresponds to 9 pm/K (linear fit; error-bars represent resolution of optical spectrum analyzer (0.02 nm)) with minor (**g**) PV change.

The sensor was installed in a test pressure vessel capable of pressurizing up to 0.40 MPa. The pressure was monitored with a manometer, accurate to ±0.02 MPa. For sensor interrogation, an unpolarized wideband Er-doped amplified spontaneous emission source was used. The signal, at approximately *λ*_*s*_ ≈ 1550 nm, was coupled through one branch of a 3 dB splitter, with the other branch connected to an optical spectrum analyzer (Yokogawa AQ6370D) with a spectral resolution up to 0.02 nm. The PV shift of the center fringe (see Fig. 7a–c) was up to 7.24 dB/MPa, with a minor hysteresis of up to 0.06 dB noticed at atmospheric pressure. Through curve fitting experimental data (see Methods), the polarimetric pressure sensitivity was determined to be $1.7\times {10}^{2}\pm 10\%$ rad·MPa⁻¹·m⁻¹. This represents an improvement in sensitivity of up to three orders of magnitude compared with circular cross-section optical fiber containing an elliptical core^37^. Although the observed hysteresis represents ~3% FSR, it is within the noise level of the measurement system and did not indicate permanent stress effects upon testing. For minimum and maximum loading conditions, it showed that the dominant frequency peak remains unchanged. This confirms that the interferometric response is narrowband and quasi-static. The Fourier-phase analysis of the sensor operation revealed a clear and monotonic dependence of the extracted phase on applied pressure with sensitivity of up to 31.6 rad/MPa (see Fig. 7d) with cross-sensitivity for temperature of 0.187 rad/K i.e., <1% of the pressure sensitivity. The linear fit (Fig. 7c) represents a section of a periodic Vernier function, while the full period could not be captured due to the maximum pressure limit of the available vessel. Based on the cavity design, the maximum measurable pressure before ambiguity occurs corresponds to one Vernier period, beyond which phase tracking would be required for unambiguous measurement. To evaluate any temperature influence on the readings, in Fig. 7e–g the thermal response of the sensor is shown. The spectral shift in temperature was in line with expectations, typical for an FBG in silica-based fibers^38^. The PV change in temperature was in the order of 8 × 10^−^^3 ^dB/K, underlining the desired low cross-sensitivity that allows for decoupling pressure sensitivity from temperature influence.

**Fig. 8:**
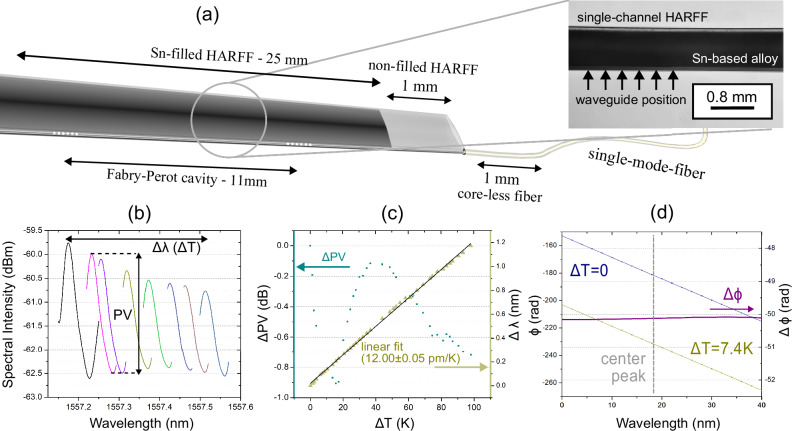
Performance of the proof-of-concept temperature sensor. Sketch (**a**) of the alloy filled sensor based on a single channel HARFF spliced with circular fiber (with consistent cross-section along the length, shown in Fig. 3**d** and the splice in Fig. 5c); the inset shows its micrograph, bottom illumination used; **b** spectral response of the center fringe in the sensor, and **c** its sensitivity demonstrated up to 12 pm/K and ±0.075 dB/K in 20 K periods. Phase response of the temperature sensor (**d**) under target stimulus, with the differential phase Δϕ. For **d**, the spectral shift was normalized to absolute wavelength values to enable accurate phase comparison.

In the proof-of-concept temperature sensor, in the sidewall of an alloy-filled, single-channel µHARFF (Fig. 3d), a laser-written waveguide with a rectangular cross-section (see *Methods*). A micrograph of the alloy-filled fiber is shown in Fig. 8a. To fill the air channel, an approximately 0.1 m section of the fiber was heated to 250 °C. One end was placed in a container with molten alloy, while the other was connected to a vacuum pump, allowing the melt to fill the channel. Subsequently, the filled HARFF was cooled down, cleaved, and spliced with commercial fiber as earlier described. As a sensing element, an FP-FBG cavity similar to the used in the twin-air-channel pressure sensor was laser-written into a side wall of µHARFF (see Fig. 8a). To demonstrate the sensor performance, it was placed on a DC-controlled heating element, while the temperature was monitored with a thermocouple. Increasing temperature induced both a visible change in fringe contrast, governed by the birefringence, and a typical spectral shift. Figure 8b shows the center fringe evolution with increasing temperature. The increasing temperatures affected the red shift as well as the change of the fringe contrast (ΔPV); both in line with the expectations. The fringe contrast showed a periodic sign change, first at around *ΔT* ≈ 13 K. In this region, the extracted FP-FBG phase of the center fringe increased linearly with temperature, with a measured sensitivity of 6 rad/K. In comparison to the twin-channel µHARFF, alloy-filled single-channel µHARFF showed a 50 % higher spectral shift of up to 12.1 pm/K. The PV temperature sensitivity was an order of magnitude higher in comparison to the sealed, twin-air-channel HARFF discussed earlier, up to ±0.075 dB/K, as shown in Fig. 8 (c).

## Discussion

In the preceding sections, the concept of the HARFF used for temperature and pressure sensing has been demonstrated. In the case of a solid HARFF, shown in Fig. 3c, the unique manufacturing approach, described in Section 2, enabled the production of an ultra-high AR of up to approximately 20:1. The solid HARFF cross section had a high flatness with maximum top-bottom surface variations of less than 1 µm, measured with an optical profiler (ZeGage™ Pro HR 3D), and shown in ref. ^39^. The initial preform AR was 33:1, limited by commercially available wafer dimensions and the aperture of the draw tower furnace. The discrepancy between ARs of the preform and the resultant fiber was in line with the expectations and may be attributed to surface tension and viscous forces in the neck-down region during the drawing. In the future, alternative drawing strategies will be explored to further increase the HARFF shape control. The measured propagation loss of ~0.16 dB/m reflects a proof-of-concept HARFF implementation rather than a fundamental limitation of the flat-fiber geometry. Loss is primarily attributed to the small, elliptical core, which increases confinement-related bend sensitivity at 1550 nm, and to residual OH⁻ absorption arising from the use of stacked XJGS1 silica wafers. Both effects are non-intrinsic, and further loss reduction is expected through core-geometry optimization and the use of low-OH infrared-grade silica. Furthermore, in the future, chemical features supporting fluorescence will be explored towards e.g., multi-channel amplification and in-HARFF signal generation.

In the proof-of-concept study, it was shown that the birefringence was enhanced in two types of sensor applications. In the case of twin-channel pressure sensors, up to three orders of magnitude improvement was achieved over commercial solid fibers, and up to two orders of magnitude improvement over other silica fibers that have been optimized towards hydrostatic pressure sensitivity^21,25,40,41^. It is noteworthy that the µHARFF sensor achieves high pressure sensitivity within a fiber-only architecture, with no reliance on external structures to enhance response. Its all-silica composition ensures mechanical robustness and stability under extreme environmental conditions. The polarimetric visibility response of the FP–FBG cavity is inherently phase-periodic due to the 2π nature of birefringence-induced phase accumulation. In the proof-of-concept study, the pressure sensor operated within the initial monotonic regime of the polarimetric response, up to approximately 0.3 MPa, beyond which phase wrapping leads to non-monotonic behavior. This operating range is sufficient for the intended demonstration and does not reflect a fundamental limitation of the architecture. If required, the unambiguous pressure range can be extended using established techniques such as multi-interferometer quadrature schemes^42^. Furthermore, the Fourier-phase method revealed a strong dependency on applied pressure, with a measured sensitivity of 31.6 rad/MPa. A level of responsivity is achieved that is significantly higher than that reported for many conventional sensors, which confirms the suitability of the µHARFF architecture for compact, high-resolution pressure sensing. Importantly, the temperature cross-sensitivity of the pressure cavity was found to be only 0.187 rad/K, representing less than 1 % of the pressure response. The small thermal contribution indicates that the cavity is largely temperature-stable, and that pressure-induced changes dominate the phase behavior over realistic operating conditions. Such low cross-coupling is especially valuable in environments where pressure measurements must remain robust despite thermal fluctuations.

The functionality of the µHARFF can be enhanced by creating a multi-material hybridization. A temperature sensor was created for the demonstration case, where a low-melting-temperature Sn-based alloy was used. The contrast of the fringes changed periodically with temperature, indicating strong birefringence induced in the waveguide. In contrast to the pressure sensor, the temperature sensor demonstrated a strong thermo-optic response with a sensitivity of 6 rad/K. This confirms that the FP-FBG structure can be engineered to support selective thermal responsivity, enabling the development of a complementary sensor channel dedicated to temperature measurement. The clear separation of sensitivities between the two types of HARFF-based sensors i.e., one pressure-dominant and the other temperature-dominant, creates an opportunity for multi-parameter sensing with low cross-sensitivity.

To increase the working range of the sensor, other alloys with higher melting temperatures could be introduced, noting that the key tailoring requirement is the difference in the CTE. It is noteworthy that the exact geometry of the µHARFF sensor can be further optimized to increase or linearize its sensitivity, by, e.g., shape optimization or increasing the metal-to-glass ratio in the cross-section; further optimization will be the object of future work. Furthermore, the simulations showing strong µHARFF birefringence response to external stimulus align well with the results provided from the proof-of-concept study.

Beyond the proof-of-concept applications, it is important to note that the HARFFs offer greater versatility for internal microstructure, as their high aspect ratio geometry offers potential for designs unavailable in traditional circular fibers, which are constrained by their axisymmetric nature. Hence, the widthwise elongated microstructure, realized through the µHARFF fabrication approach, unlocks high strain sensitivity in the plane normal to waveguide propagation direction that is challenging to attain using traditional fibers^24^. The HARFF geometry inherently enables birefringence tuning through adjustments in aspect-ratio and loading conditions, as shown in the FEM simulation. Birefringence values of the designs presented are in the order of 10^−^^5^. Further increase in birefringence sufficient to achieve polarization-maintaining properties would be possible by modifying the fiber design. The unique unidirectional rigidity of HARFF enables precise strain decoupling, making it particularly beneficial for sensing applications requiring directional sensitivity, such as measurement of internal strain, e.g., in a high-value laminated composite structure. Similarly, their conformability to laminated stacks makes them well-suited for e.g., battery condition monitoring, where pressure and temperature sensitivity is pivotal for monitoring of internal pressure build-up from electrode expansion, gas formation, or thermal events. In addition, the planar architecture and accessible surfaces facilitate direct incorporation into microfluidic and lab-on-chip systems, enabling refractive-index, pressure, and biochemical sensing within compact substrates. In the context of these sensing scenarios and among various sensing technologies, silica fibers uniquely stand as a robust, resilient, and scalable solution suitable for extreme environments. Therefore, future efforts will focus on the use of the µHARFF concept in structural health monitoring applications, noting that the present study utilizes discrete sensors, based on short sections of various HARFF, while the demonstration of distributed sensing is planned in the future. The agreement between measured mode profiles and simulations, together with low loss and clean OTDR traces, indicates a uniform and defect-free draw upon which such sensing functions rely.

In conclusion, the newly introduced high-aspect ratio flat fibers (HARFF) represent a novel type of specialty optical fibers with a non-circular, quadrilateral target cross-section. The HARFF significantly improves manual handling of thin fibers while remaining highly flexible and sensitive. The HARFF is an ultra-thin and ultra-flexible optical fiber in the thickness dimension, while offering high stiffness and structural integrity in the other dimension due to its width. Furthermore, the quadrilateral cross-section of HARFF mitigates buckling and twisting that is typical for circular fibers. The proof-of-concept study, described in the paper, demonstrated that the HARFFs are fully compatible with commercial fibers, so they can be integrated with the existing fiber systems and infrastructure. While the HARFFs demonstrated for the proof of concept are primarily designed for multiparameter sensing, the flat-fiber geometry offers potential in other specialized domains. Larger mode-area or multicore HARFF designs could enable high-power laser delivery through improved thermal coupling to a substrate, while the ribbon-like geometry may support beam-delivery or beam-combining architectures where a monolithic planar geometry is advantageous.

This work therefore, underlines the opportunities brought about by the unique geometry and structure of the HARFF. Hence, an exciting new concept for novel multi-disciplinary sensing platforms that can be leveraged in microfluidic, fiberized integrated planar photonic devices, ultra-sensitive distributed sensing, power scaling, and multiplexing has been presented.

## Method

### FEM simulation and parametric study

The simulation was run using Abaqus FEA, Dassault Systèmes. The µHARFF cross-section with two air-channels was defined using the parameters in Fig. 2a; width ($w$), height ($h$), wall thickness ($t$), and microstructure spacing ($\delta x$). This shape was used to create a simple plane (2D) strain linear elastic model using CPE4R elements, which simulated hydrostatic pressure by applying 0.1 MPa and 0.4 MPa to the internal and external surfaces, respectively. A Young’s modulus of 73 GPa and a Poisson’s ratio of 0.2 were used for silica glass material properties. The parametric µHARFF geometries were generated by systematically varying the geometric parameters $w$, $h$, $t$, and $\delta x$, producing fiber cross-sections with different aspect ratios and wall thicknesses. Each parameter was varied independently over a defined range ( ± 50%) about the baseline values (Fig. 2a) using five discrete intervals.

### CO_2_ laser-based preform manufacturing

The system comprised a CO_2_ laser (Universal Laser Systems, λ = 10.6 µm) with a maximum output power of 60 W and a cartesian (x-y-z) motion control unit governed via RepRap firmware. The CO_2_-laser-based heating enables rapid and localized melting of silica-based glass as outlined in ref. ^16^. The CO_2_ laser beam was shaped through an interchangeable ZeSe lens system to provide a numerical aperture (NA) of the incident beam that can be tuned in the range of 0.05–0.10.

### Materials

The fibers were drawn using preforms that were in-house assembled and laser-processed. To obtain a preform, fused silica wafers (XJGS1) and germanium-doped core-cladding, silica rods (GIF625, Thorlabs Inc) with the cladding diameter etched to approximately 80 µm and the core of 62.5 µm (n_core_ ≈ 1.4749, at λ = 1300 nm), were used. In the following experiments, the assembled preforms had a nominal width of 33 mm, which was dictated by the maximum aperture of the available draw-tower furnace. In the case of demonstrated proof-of-concept fiber sensors, a short section of drawn HARFF was spliced with a commercial counterpart: (a) single-mode fiber (SMF-28e + , Corning Inc) or (b) coreless termination fiber with a diameter of 125 µm (FG125LA, Thorlabs Inc.). The sensors were tested following approximately a 10-month ageing cycle, showing repeated characteristics. The tests involved repeated heating and cooling cycles. Within the range investigated, no evidence of progressive degradation was detected. The higher coefficient of thermal expansion of the alloy relative to silica is expected to generate compressive interfacial stresses during heating, helping to maintain interfacial contact.

### Splicing of HARFFs

HARFF was spliced with commercial fibers using a laser-splicer (LZM-100, Fujikura Ltd) as shown in Fig. 5a. The splicer was equipped with a custom-made holder that enabled 5-axis alignment of the HARFF with respect to the circular fiber. In comparison to a standard fiber holder, i.e., v-groove, a 1 mm wide flat-bottom slot was machined in a 2 mm diameter brass rod. A cleaved HARFF/µHARFF was secured in the groove using 1 mm-thick rubber gasket, and then it was placed in a standard detachable 2 mm endcap holder (Fujikura FH-100-2000). The splicing recipe comprised: (i) initial alignment of the circular fiber to the desired position on HARFF with a gap of 10 µm, (ii) simultaneous melting of both fibers by an incident laser beam, (iii) contacting the molten fibers with an overlap of 3 µm, and then (iv) tapering of the splice (10 µm). The splicing parameters were fine-tuned, within original firmware boundaries, depending on HARFF geometry and splice specification as explained in Section 2. The splice loss as estimated via HARFF transmitted power measured before and after splicing with a short (60 cm long) µHARFF section, using drops of high-index oil to reduce the impact of cladding light.

### Laser-writing of fiber Bragg gratings

The fiber Bragg gratings were written using a femtosecond laser writing system^43^ consisting of a Yb:KGW laser (*λ* = 1030 nm, 200 fs pulses at 200 kHz, PHAROS, Light Conversion) that was frequency doubled, resulting in a *λ* = 515 nm second harmonic inscription beam. A quarter waveplate was used to produce circular polarization to ensure a smooth refractive index change while mitigating nanograting damage formation, which could lead to high polarization-dependent losses^44^. The beam was focused using a ×50 immersion objective of variable NA 0.5-0.9 (PLN50XOI, Olympus) into the µHARFF, using an *n* = 1.53 immersion oil (Laser Liquid, Cargille). During writing, the objective NA was set to 0.9, and each point was written by firing a single pulse (*E*_*p*_ ~ 90 nJ). A period of 2.1 µm was chosen, which corresponds to a 4th-order *λ* = 1550 nm grating.

### Waveguide writing

In the case of coreless HARFF (e.g., Fig. 3c, d) a short section of coreless fiber (FG125LA, Thorlabs Inc) was used as an intermediate splice to accommodate small alignment tolerances between the core and the thin-wall of HARFF. Subsequently, a rectangular cross-section waveguide 10 by 8 µm, was laser written in µHARFF by rastering 50 scanlines at 200 nm lateral separation in a multiscan pattern^45^ with a scanline pulse density, *D* = 3×10^5^ pulses/mm, pulse energy *E*_*p*_ = 50 nJ and objective set to 0.5 NA. Based on previous work^43^, this induces an estimated index change *Δn* ~ 3×10^−^^3^. Note that waveguide birefringence arises not only from the rectangular geometry, but also from laser-induced local strain anisotropy. It was estimated to be 1.678 × 10^−5^, based on the spectra shown in Fig. 8a.

### Birefringence and polarimetric pressure sensitivity extraction

Polarimetric pressure sensitivity was extracted from the FP-FBG spectral response by curve-fitting to the evolution in birefringence, subject to pressure, *P*. Here, the pressure response arises from a stress-induced birefringence modification within the cavity, which changes the cavity finesse and therefore the fringe contrast. The observed signal is thus an intensity modulation associated with a change in interference pattern visibility. The curve-fitting routine exploits the fact that applied pressure induces a differential phase delay between two orthogonally polarized eigenmodes, leading to a measurable modulation in the FP-FBG interference fringe visibility.

The FP-FBG reflection spectrum was fitted as the superposition of two orthogonally polarized components corresponding to the fast and slow axes of the fiber. Each spectral signature component was assumed to have equal amplitude and an identical spectral envelope, consistent with a zero-birefringence reference, yielding maximum empirical fringe visibility at zero applied pressure. Applied pressure was assumed to introduce a differential wavelength separation, *Δλ*, between these two components, corresponding directly to a change in phase birefringence, *ΔB*. The experimentally measured variation was fitted by adjusting *Δλ* using a Nelder–Mead optimization routine, minimizing the least-squares error between the measured and modeled spectra. The corresponding polarimetric pressure sensitivity, *K*, was then calculated as

3 $$K=\frac{2\pi }{{\lambda }_{0}}\frac{\partial B}{\partial P}$$

### Fourier-phase analysis of FP-FBG interferometric signals

To evaluate the pressure and temperature responses of the FP-FBG cavities, Fourier-phase analysis was applied to the reflected interferometric spectra. Reflected spectra were recorded using an optical spectrum analyzer (OSA). Spectra were normalized to compensate for source fluctuations. An FTT was then applied to obtain the complex spatial-frequency. The dominant peak was filtered, and the phase component for the center fringe (see Fig. 7a, e and Fig. 8b) was unwrapped from IFFT spectra (see Fig. 7d and Fig. 8d). The sensors were subjected to stimuli 0-0.2 MPa and ΔT_p_ 50 K, and ΔT_t_ 7.4 K for pressure and temperature sensor, respectively, where ΔT_t_ represents the first sign change for ΔPV (see Fig. 8c). The resultant phase shifts $(\Delta {{{\rm{\phi }}}})$ were fitted against the stimuli using a linear fit. Cross-sensitivity was determined by measuring phase changes under the *non-target* measurand.

## Supplementary information

Transparent Peer Review file

## Data Availability

The methodology and Python scripts used for generating and meshing the parametric geometry, performing the mesh sensitivity study, and conducting the parametric analysis are provided in the Zenodo database under 10.5281/zenodo.18985925.

## Code availability

The methodology and python scripts for generating/meshing the parametric geometry and for performing the mesh sensitivity study are provided in the Zenodo database under 10.5281/zenodo.18985925.
