# Supplementary material for: High aspect-ratio quadrilateral flexible silica optical fibers for enhanced multi-parameter sensing
Source: Nat Commun. 2026 Apr 29;17:5636. doi: 10.1038/s41467-026-72416-6 (PMC13315245; doi:10.1038/s41467-026-72416-6)
Supplement: Supplementary file 1 — Transparent Peer Review file [file 41467_2026_72416_MOESM1_ESM.pdf]

nature portfolio

# Peer Review File

# High aspect-ratio quadrilateral flexible silica optical fibers for enhanced multi-parameter sensing

Corresponding Author: Dr Pawel Maniewski

**This file contains all reviewer reports in order by version, followed by all author rebuttals in order by version.**

Version 0:

Reviewer comments:

Reviewer #1

(Remarks to the Author)
This paper presents a high-aspect-ratio optical fiber fabricated using laser processing and the stack-and-draw method, and explores its applications in pressure and temperature sensing. From the perspective of fiber structure, this work demonstrates a certain degree of innovation, which could potentially expand the functionality and application scenarios of existing fiber optic sensors.

However, this study has several limitations. As a novel optical waveguide, it lacks systematic investigation into the optical guiding properties, such as transmission loss, mode characteristics, and spectral bandwidth. Furthermore, the two sensing applications demonstrated in this work do not exhibit superior sensitivity compared to existing technologies, and the paper fails to provide a comprehensive performance comparison with current fiber optic sensors.

In conclusion, the work presented in this paper falls short of the innovation and systematic rigor required for publication in Nature Communications. The authors are advised to further refine the characterization of the fiber's optical performance and develop more compelling sensing applications to strengthen their findings.

(Remarks on code availability)

Reviewer #2

(Remarks to the Author)
Fiber-optic pressure sensors have been extensively studied and widely employed in various critical applications, including the oil and gas industry. Conventional sensors typically incorporate well-designed transducers and specialized material packaging to ensure pressure sensitivity, stability, and reproducibility across a wide pressure range while mitigating temperature crosstalk. The present work demonstrates an innovative approach by integrating these functionalities directly into the fiber itself, which is commendable. However, for robotic pressure sensing applications, several concerns regarding the proposed sensor remain unaddressed:

1. Poor spectral response to pressure: As evident in Figure 6a, the broadband spectral profile undergoes significant distortion under applied pressure. This substantial shape variation suggests that optical intensity-based pressure quantification would suffer from irreproducible fluctuations.
2. Reproducibility issues: The splicing connection shown in Figure 5 (inset) appears rather crude. Such fabrication quality falls short of the established standards seen in commercial pressure sensors, which demonstrate well-optimized manufacturing processes and consistent sensing performance.
3. Insufficient polarization analysis: Like most fiber pressure sensors, the proposed design exhibits strong polarization dependence. A more thorough discussion is needed regarding: (a) system response to different input polarization states, and (b) birefringence effects induced by the twin FBGs inscription process.
4. Lack of clarity on alloy-filled sensor: Figure 7 fails to provide sufficient details about: (a) the microstructure fiber's functionalization mechanism, (b) corresponding broadband spectral characteristics, and (c) the splicing methodology between alloy-filled fiber and coreless fiber.
5. Overlooked advancements in flat fiber technology: The past decade has seen significant progress in flat fiber

development, such as the slab fiber (F150408B K7s) fabricated by Prof. Gangding Peng's group at UNSW. While adapting this platform to twin-channel architecture represents notable progress, critical challenges remain in achieving the stability, reproducibility, and robotic compatibility that conventional interferometric structures with simpler configurations already offer.

(Remarks on code availability)
None

Version 1:

Reviewer comments:

Reviewer #1

(Remarks to the Author)
After reviewing the revised manuscript, I note that there remains an absence of content addressing two critical aspects: a discussion on the fiber's optical guiding properties and an elaboration of its potential for high-value sensing applications. Notably, practical application scenarios for fiber optic sensing impose stringent requirements on two core performance metrics of fibers: consistency (uniformity of key parameters across the fiber length/batch) and robustness (mechanical stability, environmental resistance, and long-term reliability). Regrettably, the HAREF fiber proposed in this paper appears unlikely to meet such demanding criteria for real-world deployment.

Reviewer #2

(Remarks to the Author)
Although the authors have responded to my previous questions and made some revisions to the manuscript, I still believe that this work has not met the high publication standards of Nature Communications. After a careful review, I still have some questions and concerns, which are described as follows:

1. Lack of Substantial and Clearly Demonstrated Novelty: The manuscript positions HARFF as a novel platform. However, the core manufacturing techniques (laser welding and stack-and-draw) have been previously reported by the same group and others for circular and non-circular fibers [16, 31, 33, 34]. The advancement seems to be an application of existing methods to a quadrilateral shape rather than a foundational innovation. A direct and quantitative comparison with state-of-the-art flat or micro-structured fibers from the literature (e.g., [24, 25, 28, 30, 39, 40]) in terms of sensitivity, form factor, and manufacturing complexity is missing, making the claimed "three orders of magnitude improvement" seem unsubstantiated and potentially misleading.

2. Unconvincing and Poorly Explained Sensing Mechanism: The fundamental working principle of the sensors is unclear. The manuscript introduces birefringence (Eq. 1) as the key mechanism but measures the Peak-to-Valley (PV) power change in a Fabry-Perót cavity, which is an intensity-based signal. The critical physical link between the induced birefringence and the modulation of the FP fringe contrast is not rigorously derived or explained. Is it due to a change in the finesse, polarization-dependent loss, or modal interference? This lack of clarity undermines the scientific soundness of the work.

3. Inappropriate and Misleading Performance Metrics: The use of dB/MPa and dB/K as primary sensitivity metrics is highly unconventional and problematic for a sensor based on FBG/FP structures. Intensity-based measurements are inherently susceptible to source fluctuations and external perturbations, making them unreliable for practical applications. The standard, robust metric for such sensors is wavelength shift (pm/MPa, pm/K). The authors' choice to emphasize the unstable intensity signal raises concerns that the actual wavelength sensitivity may not be competitive. The comparison justifying performance enhancement is invalid, as it conflates resolution (kPa) with wavelength sensitivity (pm/MPa).

4. Significant Technical Hurdles Ignored and Lack of Practicality: The manuscript promotes scalability but demonstrates only discrete, short-length sensors that require extensive and complex post-processing: $CO_2$ laser sealing of microstructures, post-draw alloy filling, and femtosecond laser inscription of waveguides and gratings. These processes are not scalable to long lengths and are incompatible with the proposed vision of distributed sensing. The practical feasibility of this platform for real-world applications is therefore highly questionable.

5. Critical Lack of Experimental Rigor: The performance data are presented without any statistical validation. Key parameters like sensitivity are reported from single measurements without error bars, standard deviations, or results from multiple sensor samples. This makes it impossible to assess the reliability and reproducibility of the findings.

6. Incomplete and Unprofessional Manuscript Preparation: The manuscript is not ready for peer review. Numerous placeholder errors ("Error! Reference source not found.") and inconsistent figure citations severely disrupt the reading flow and reflect poorly on the care taken in its preparation. The methodology section lacks essential details for reproducibility, such as FEM boundary conditions and the complete set of laser writing parameters.

In conclusion, while the HARFF geometry itself may have potential, this manuscript fails to present a compelling, novel, and scientifically rigorous case for its superiority as a sensing platform. For these reasons, I do not recommend that this manuscript be published in Nature Communications.

Version 2:

Reviewer comments:

Reviewer #1

(Remarks to the Author)
The authors have provided a comprehensive response to my questions, and I believe the revised manuscript is suitable for publication in Nature Communications.

Reviewer #3

(Remarks to the Author)
Reviewer Report
Manuscript Number:
Title: High aspect-ratio quadrilateral flexible silica optical fibers for enhanced multi-parameter sensing
Overall Recommendation: Minor Revision
General Assessment
The manuscript reports a new planar silica optical fiber platform, characterized by an ultra-high aspect ratio is introduced, termed High Aspect Ratio Flat Fiber (HARFF). With a quadrilateral cross-section, HARFFs have enhanced mechanical properties compared to typical optical fibers for the purpose of multiparameter sensing. Based on the laser- based processing combined with stack-and-draw fabrication, HARFFs possess more flexibility in terms of mechanical properties and microstructure, which can be designed to be solid fiber, air-channel, single core and multi-core, paving the way for diverse applications. Numerical simulation is performed to precisely adjust the parameters of the fiber, pressure and temperature sensing experiments are demonstrated after the fabrication and characterization of this fiber. Moreover, the authors provide a perspective on the future application and optimization potential of this optical fiber.
Overall, the manuscript is clearly written and well organized, possessing an important impact on the academic communities of special fiber fabrication and fiber sensor, and I believe this work would be of interest to readers and suitable for publication provided that a few issues can be addressed.
Comments
1. By precisely controlling the structural parameters of the optical fiber, the author adjusted its birefringence. Can this HARFF be designed to have polarization-maintaining properties?
2. In the case of single- and multi-core μHARFF a transmission loss of approximately 0.16 dB/m at $\lambda$ = 1550 nm is estimated using cut-back technique. The loss of HARFF can be further reduced? In its fabrication process, which step is the primary factor limiting its loss? With continuous future optimization, will this fiber become comparable to conventional fiber in terms of transmission loss?
3. HARFFs have enhanced mechanical properties compared to typical optical fibers for the purpose of multiparameter sensing. Therefore, the author primarily addresses the application and future development of HARFF in the field of sensing. Apart from sensing applications, can this HARFF be utilized in fields such as optical communication or laser power delivery? If feasible, what specific structural or process optimizations would be required? Conversely, if its application is challenging in these areas, what are the primary limiting factors?
4. Subfigure (b) in Fig. 4 is missing.
5. Figure 6 demonstrates the splicing of HARFF with a conventional optical fiber. However, the author does not provide the corresponding splicing loss. The author achieved stable splicing by experimentally adjusting the splicing parameters. Given the novel structure of the fiber discussed, does achieving low-loss splicing necessitate a complex optimization process? Furthermore, for HARFF with a higher aspect ratio, does splicing with conventional single-mode fibers become significantly more challenging?
6. The Discussion section could be made more concise. Some statements have been repeated within this section and appear elsewhere in the manuscript.
7. The authors claim an improvement of "up to three orders of magnitude" in sensitivity. However, the manuscript compares a customized intensity-based metric (7.24 dB/MPa) against traditional wavelength-shift metrics. To validate this claim, please provide a calculation converting the dB variation into an equivalent refractive index change or strain resolution, allowing for a direct physical comparison with standard FBG sensors.
8. A hysteresis of 0.06 dB is reported. Given the peak-to-valley sensitivity, this represents a non-negligible error (approx. 3% FS). Could the authors discuss the origin of this hysteresis? Is it related to the polymeric coating or the residual stress at the $CO_2$ laser-sealed ends?
9. In Fig. 9(c), the PV response to temperature is periodic. Please clarify the monotonic dynamic range of the sensor. For the pressure sensor, does the linear fit in Fig. 8(c) represent a small section of a similar periodic Vernier function? What is the maximum measurable pressure before ambiguity occurs?
10. Long-term Stability of Metal-Filled Fibers: For the Sn-alloy filled sensor, did the authors perform any thermal cycling tests? Please comment on the potential delamination risks at the silica-metal interface due to the large CTE mismatch over repeated cycles.

Reviewer #4

(Remarks to the Author)
The authors have presented the fabrication of a new type of optical fibre that features high aspect ratio. The preform

preparation is the unique feature, whereby two wafers suspended by a silica rod are used. The preform can be pressurised to create different geometries such as either hollow or solid ribbon fibres. The central argument for this use is sensing, particularly hydrostatic pressure sensing through birefringence measurement. They also demonstrate temperature sensing by filling with a metal alloy.

The paper appears to be mostly technically sound, particularly on the fabrication elements. Unfortunately, the sensing results are quite underwhelming. The pressure response (Fig. 8a) seems too weak to be able to measure a wavelength shift. The temperature response is quite similar to conventional SMF28 FBGs (~10pm/K). For this reason, I do not believe the impact will be sufficient to justify publication in Nature Communications. I provide the following feedback to assist the authors in improving their paper.

1. In regard to the pressure sensitivity I will make a comparison to conventional single mode fibre, which has a hydrostatic pressure sensitivity of approximately 10^-11 strain/m. Value from Foster JLT, 39, 2514 (2021). This would lead to an FBG sensitivity of 16 pm/MPa. If the result in the author's paper is correct, they should have a sensitivity three orders of magnitude higher, at 16 nm/MPa. This should be easily resolvable using the author's measurement, but clearly is not. My rough estimate from the author's data is that they have a sensitivity of 8 pm/MPa, even less than standard fibre.
2. The authors may benefit from performing Fourier phase analysis of their signals, which is a common approach for fibre optic interferometry.
3. Fig. 1: Where does the data come from? Also, what are the two dots on the graph?
4. I have a query relating to the numerical results in Fig. 2. The authors give Eq. 1 which relates birefringence directly to the difference in the principal components of strain in the transverse plane. To my understanding, this intrinsically assumes that the principal component directions are aligned (everywhere) to the cartesian grid. I do not believe this would be the case due to the complex geometry. The local refractive index components would need to be evaluated considering the variation of principal strain directions across the geometry, before then calculating birefringence. Further, it would be good to go a step further and calculate the birefringence of the optical modes and compare these values to the experimental results.
5. Line 157: not clear what "high confinement" means.
6. Line 163: more detail needed ab out the germanium-doped fused silica core rods. The diameter? Are they uniformly doped, or core clad?
7. Line167: what laser wavelength is used? I see it's in the methods section. I believe directly referencing to the methods section could be used in the main text as some information is provided in the main text, but not all, making it a bit confusing.
8. Fig. 4: It's not quite clear what the image with "weld" is showing. Is this the edge of the two-channel fibre?
9. Fig. 5c: the colour bar tick labels are quite small and the image seems to be low resolution.
10. Splice losses should be mentioned with respect to Fig. 6.
11. The referencing to Fig. 6c and Fig. 6b seems to be incorrect in lines 221 and 223.
12. Line 269: I believe this should be 10^-2 dB/K.
13. Fig. 8c: Why are there only errors bars for the depressurizing results? What are the error bars representing?
14. Fig. 9a: Why does the fibre appear to be tapered?
15. Fig. 9c: colour code the y-axes to the data, maybe also add arrows so that it's clear which axes the two sets of data correspond to.
16. Line 382: coreless fibre is mentioned but I can't see where this was used in the main text (ignore if I just missed this).
17. There are many grammar errors throughout the paper, some examples:
a. Unnecessary commas on line 26
b. Lines 26-29, sentence is too long
c. Unnecessary comma on line 40
d. Missing comma after waveguide on line 45
e. Line 65, "Fibres" should be "Fibre"
f. Capitalisation is used when acronyms are defined, which is unneceesary (except proper nouns)
g. Line 81, "HARFF pressure sensor can achieve sensitivity a resolution of …" needs to be corrected
h. Line 156, "The HARFF preforms utilize a custom", odd language to say that the preform "utilizes" something
i. Line 279, "As a sensing element…"
j. Line 317: "This was made possible…"

Version 3:

Reviewer comments:

Reviewer #3

(Remarks to the Author)
The author has addressed those specific concerns. I believe the manuscript, in its current form, is suitable for publication in the Nature Communications.

Reviewer #4

(Remarks to the Author)
The authors have satisfactorily addressed the technical aspects of my previous review.

I still believe that the pressure sensitivity results are inadequate to justify publication in Nature Communications. The results

in Fig. 7c are based on intensity changes rather than a wavelength shift, which is due to the wavelength shift being quite small. This indicates that the intrinsic sensitivity is low and the observed intensity changes could quite easily come from other factors affecting light transmission. In any case, the error bars indicate a sensing resolution the order of 0.1 MPa, which only has limited applications.

Version 4:

Reviewer comments:

Reviewer #4

(Remarks to the Author)
The authors have provided detailed responses to my comments. I believe the revised manuscript is suitable for publication in Nature Communications.

**Open Access** This Peer Review File is licensed under a Creative Commons Attribution 4.0 International License, which permits use, sharing, adaptation, distribution and reproduction in any medium or format, as long as you give appropriate credit to the original author(s) and the source, provide a link to the Creative Commons license, and indicate if changes were made.
In cases where reviewers are anonymous, credit should be given to 'Anonymous Referee' and the source.
The images or other third party material in this Peer Review File are included in the article's Creative Commons license, unless indicated otherwise in a credit line to the material. If material is not included in the article's Creative Commons license and your intended use is not permitted by statutory regulation or exceeds the permitted use, you will need to obtain permission directly from the copyright holder.
To view a copy of this license, visit https://creativecommons.org/licenses/by/4.0/

Dr Pawel Maniewski
KTH Royal Institute of Technology
Stockholm
Sweden
pawelma@kth.se

Dear Reviewers,

Following editorial advice, I am pleased to share our revised manuscript entitled "***High aspect-ratio quadrilateral flexible silica optical fibers for enhanced multi-parameter sensing***" for consideration as a Research Article in Nature Communications. In the revised manuscript and in the following, we have addressed the points raised by the reviewers of our original submission and highlight changes in the revised manuscript for your consideration. For clarity, the reviewers' comments are marked **blue** and our comments in **black**. In the end of this letter, a revised manuscript with changes highlighted in yellow is shown.

***Reviewer #1 (Remarks to the Author):***

*This paper presents a high-aspect-ratio optical fiber fabricated using laser processing and the stack-and-draw method, and explores its applications in pressure and temperature sensing. From the perspective of fiber structure, this work demonstrates a certain degree of innovation, which could potentially expand the functionality and application scenarios of existing fiber optic sensors.*

*However, this study has several limitations. As a novel optical waveguide, it lacks systematic investigation into the optical guiding properties, such as transmission loss, mode characteristics, and spectral bandwidth.*

In the original manuscript we focused on optomechanical advantages only, unlocked by the innovative high-aspect ratio design and microstructure, as these have not been reported. On reflection, we agree with the reviewer, so we have revised the manuscript to include a detailed optical performance analysis of waveguides, which does full justice to our novel work, see Pages 3-4 in the revised manuscript.

*Furthermore, the two sensing applications demonstrated in this work do not exhibit superior sensitivity compared to existing technologies, and the paper fails to provide a comprehensive performance comparison with current fiber optic sensors. In conclusion, the work presented in this paper falls short of the innovation and systematic rigor required for publication in Nature Communications. The authors are advised to further refine the characterization of the fiber's optical performance and develop more compelling sensing applications to strengthen their findings.*

In cases where existing fiber constructions rely on multi-component assemblies, using e.g., flexible, plastic bobbins with wound optical fiber, or not scalable and less robust sensors that functionalize only fiber tips we concur with the reviewer that these are able to achieve, e.g., high pressure sensitivity through deformation of the bobbin. In contrast, our approach leverages the fiber design itself only, i.e. the inherent sensitivity resulting from the unique structural design. For clarity, we have now referenced several studies showing that HARFF represents significant, up to three orders of magnitude,

improvement over other inherent silica-based fiber sensitivity, namely [18-22,29] in the revised manuscript.

***Reviewer #2 (Remarks to the Author):***

*Fiber-optic pressure sensors have been extensively studied and widely employed in various critical applications, including the oil and gas industry. Conventional sensors typically incorporate well-designed transducers and specialized material packaging to ensure pressure sensitivity, stability, and reproducibility across a wide pressure range while mitigating temperature crosstalk. The present work demonstrates an innovative approach by integrating these functionalities directly into the fiber itself, which is commendable. However, for robotic pressure sensing applications, several concerns regarding the proposed sensor remain unaddressed:*

Existing fiber-optic pressure sensors often rely on multi-component architecture such as transducers and specialized packaging (e.g., wound bobbins) to achieve sensitivity and stability. On the contrast, the enhanced sensitivity achieved in our work is inherent to the fiber and obtained using robust fused silica, thus has major benefits for structural integration. Our HARFFs have a smaller volume, which therefore reduces any risk of deleterious effects on the structural performance, and further benefits accrue where available volume is fundamentally limited.

1. *Poor spectral response to pressure: As evident in Figure 6a, the broadband spectral profile undergoes significant distortion under applied pressure. This substantial shape variation suggests that optical intensity-based pressure quantification would suffer from irreproducible fluctuations.*

The reviewer may have misinterpreted the spectra. It should be noted that it was given in log scale, with distortions visible at levels of under -70 dBm and likely linked to the background noise. Hence, in the revised manuscript we have used linear scale, underscoring that the spectra is stable and reproducible during sensor operation, see Fig. 8 in the revised manuscript.

2. *Reproducibility issues: The splicing connection shown in Figure 5 (inset) appears rather crude. Such fabrication quality falls short of the established standards seen in commercial pressure sensors, which demonstrate well-optimized manufacturing processes and consistent sensing performance.*

To conduct the pressure tests, it was necessary to seal the optical fiber ends, a resulting “tapering” process was therefore implemented. This is a reproducible technique conducted on a commercial laser splicer (Fujikura LZM-100). The tapering process deforms the ends of the HARFF, purposefully to seal. In our revised manuscript we have also included micrographs of other non-sealed splices for other to demonstrate consistent splicing without a tapered seal (Fig. 6). Further, a more detailed description on the splicing technique has been included in Methods in the revised manuscript.

It reads: <u>*Splicing of HARFFs:*</u> *HARFF was spliced with commercial fibers using a laser-splicer (LZM-100, Fujikura Ltd) as shown in Fig. 6 (a). The splicer was equipped with a custom-made holder, that enabled 5-axis alignment of the HARFF in respect to the circular fiber. The splicing recipe comprised: (i) initial alignment of the circular fiber to the desired position on HARFF with the gap of 10 µm, (ii) simultaneous melting of both fibers by an incident laser beam, (iii) contacting the molten fibers with overlap of 3 µm and then (iv) tapering of the splice (10 µm).*

3. *Insufficient polarization analysis: Like most fiber pressure sensors, the proposed design exhibits strong polarization dependence. A more thorough discussion is needed regarding: (a) system response to different input polarization states, and (b) birefringence effects induced by the twin FBGs inscription process.*

We agree with the reviewer, hence a detailed optical performance analysis is now included in the revised manuscript, which also addresses the comments of reviewer #1, see Methods in the revised manuscript. It reads: *Note that waveguide birefringence arises not only from the rectangular geometry, but also from laser-induced local strain anisotropy. It was estimated to be 1.678 × $10^{-5}$, based on the spectra shown in Fig. 9 (a).* For the sensor operation an unpolarized light was used to leverage Vernier effect.

It reads: *When unpolarized light was launched into the cavity the summation of the generated spectra can be used to infer changes of birefringence, by means of a Vernier effect [35] (Page 13)*

4. *Lack of clarity on alloy-filled sensor: Figure 7 fails to provide sufficient details about: (a) the microstructure fiber's functionalization mechanism, (b) corresponding broadband spectral characteristics, and (c) the splicing methodology between alloy-filled fiber and coreless fiber.*

We agree, so further details have been added to the revised manuscript.

In Page 8 of the revised manuscript it reads: (…)*in the case of enhanced temperature measurement, to increase sensitivity and maximize birefringence response, the single-channel μHARFF was filled, post-draw, with Sn-based alloy ($T_{melting}$ ≈ 220 °C). Here, it is more straightforward to visualize how the enhanced sensor response is generated. The functionality is based on the mismatch of the coefficients of thermal expansion (CTE) of the silica glass and the metal alloy (approximately* $0.5 \times 10^{-6}\ K^{-1}$ *for the glass and* $23.0 \times 10^{-6}\ K^{-1}$ *for an Sn-alloy). Conceptually, the metal alloy has a much greater CTE and will be constrained by the glass. The resulting misfit will generate both radial and tangential stresses in the glass and create a change in birefringence that is proportional to any temperature change. The induced stress modulates the birefringence in the glass, enabling a thermally tunable sensor. The thermal expansion will be greatest across the width of the μHARFF, hence the optimal position of the waveguide is evident.*

*5. Overlooked advancements in flat fiber technology: The past decade has seen significant progress in flat fiber development, such as the slab fiber (F150408B K7s) fabricated by Prof. Gangding Peng's group at UNSW. While adapting this platform to twin-channel architecture represents notable progress, critical challenges remain in achieving the stability, reproducibility, and robotic compatibility that conventional interferometric structures with simpler configurations already offer.*

We cannot find any reference in the literature using available search engines as shown in the images below (Fig. 1). We are aware of Prof. Peng's work at UNSW towards creating specialty fibers, but when searching 'slab fibers' by pairing his name with the 'slab/flat/rectangular/F150408B K7s fiber' phrases, but we did not find any published papers. The work the reviewer refers to may have been presented at a conference with no archival record or in private communications, so we are unable to cite and refer appropriately to this work.

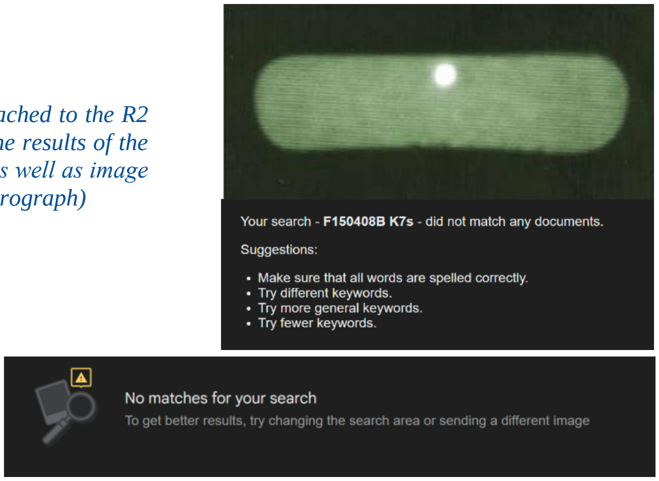


*Figure 1 The micrograph attached to the R2 review (top) and search engine results of the 'slab fiber' (F150408B K7s as well as image search using the attached micrograph)*

Furthermore, we have studied the fiber micrograph shared by reviewer #2 (Fig. 1), and have concluded that this is similar to that expected from a traditional single-mode fiber preform which has been machined to obtain a flat cross-section. This fabrication process is significantly different, more time-consuming and less scalable and less flexible (e.g., no microstructure) to the HARFFs described in our manuscript. Moreover, due to the lack of microstructure, it will not have the optomechanical advantages of the HARRF introduced in our manuscript. The authors have made comprehensive references (see Page 1) and kindly request any further specific papers be identified if their inclusion is to be considered. It must be stressed, and perhaps obvious, that the micrograph included deviates significantly from the advances made in the manuscript.

We thank the reviewers for their constructive comments and valuable discussion, which guided us towards improvements and more complete manuscript.

On behalf of all the authors,

Pawel Maniewski, PhD

We thank the reviewer for taking the time to undertake a second evaluation of our manuscript. We appreciate their constructive comments and helpful suggestions, which have further strengthened the paper. We have addressed all remaining points carefully, and we trust that the revised version now meets the expectations of the reviewer and the journal.

For clarity, in below response the reviewer's original comments are provided in **blue** and response from the authors in **black**.

Reviewer #1 (Remarks to the Author):

After reviewing the revised manuscript, I note that there remains an absence of content addressing two critical aspects: a discussion on the fiber's optical guiding properties and an elaboration of its potential for high-value sensing applications.
Notably, practical application scenarios for fiber optic sensing impose stringent requirements on two core performance metrics of fibers: consistency (uniformity of key parameters across the fiber length/batch) and robustness (mechanical stability, environmental resistance, and long-term reliability). Regrettably, the HAREF fiber proposed in this paper appears unlikely to meet such demanding criteria for real-world deployment.

We have submitted our original revised manuscript with the original highlighting in yellow, and highlighted additional text in response to this set of reviewer's comments in green. We have provided in the following our response to the reviewer's two main aspects:

1) a discussion on the fiber's optical guiding properties

Following the reviewer's original comments we have included the fibre's optical guiding characteristics, which has strengthened the manuscript. In our response we added information to section 2 (see Page 7) of the previously revised manuscript, which incorporated the near-field mode pattern in Fig. 5c (1550 nm), the numerical aperture (0.13±0.02) is stated in the text, and effective refractive index of the chemically doped waveguide ($n_{eff}$ = 1.4633). We also described in the text on Page 7 cut-back measurements over a 15 m long section of a µHARFF carried out at 1550 nm that quantify transmission loss as 0.16 dB/m. Furthermore, we described an OTDR analysis conducted to assess draw quality and identify any significant core discontinuities, which we stated (see Page 7) "*did not reveal core discontinuities or noticeable scattering centers throughout the wound fiber*".

We consider our amendments in the previous revision to constitute 'a discussion on the optical guiding properties'. However, we did not state, as it is well established in the optical fibre industry, that the Ge-doped fused silica core rods we used to manufacture the HARFFs produce no additional colour centres or absorption bands that require specific evaluation. Therefore, we have added an additional sentence on Page 7 for clarification purposes. To provide additional emphasis on the quality of the HARFF we have added the following text in the Discussion section of the manuscript Page 12 Line 17-19.

> '*The agreement between measured mode profiles and simulations, together with low loss and clean OTDR traces, indicate a uniform and defect-free draw upon which such sensing functions rely.*'

2) elaboration on the potential for high value sensing scenarios.

We appreciate the reviewer's request for further clarification. Our manuscript reports research within a £2.2M EPSRC-funded collaboration involving five universities and several industry partners targeting high-value applications. The consortium aims to integrate HARFF fibres into advanced composite structures; this work represents the foundational steps toward that goal.

The composites sector is currently valued at $133 billion with a 9.2% CAGR, and the potential of 'flat-form' optical fibre sensors for such applications is reviewed in detail in reference [22] (paragraph 3 of the introduction). In this study, we successfully demonstrate microstructure quadrilateral fibres which previously were only a theoretical concept. Upon this publication, HARFF can be integrated in future composite parts produced using realistic mould tools, a description of which may be the object of future publications. While integration depends on additional parameters beyond fibre basic performance or manufacturing route, we feel these aspects lie outside the intended scope of the present work.

Our 'proof of concept' study of pressure and temperature sensing has several applications also discussed in the manuscript. Furthermore, we have expanded the Discussion (paragraph 4) to highlight 'beyond proof-of-concept' scenarios and added examples of high-value composite structures.

Text Added, Discussion:

> *(…), e.g. in a high value laminated composite structure. Similarly, their conformability to laminated stacks makes them well-suited for e.g., battery condition monitoring, where pressure and temperature sensitivity is pivotal for monitoring of internal pressure build-up from electrode expansion, gas formation, or thermal events. In addition, the planar architecture and accessible surfaces facilitate direct incorporation into microfluidic and lab-on-chip systems, enabling refractive-index, pressure, and biochemical sensing within compact substrates. In the context of these sensing scenarios and among various sensing technologies silica fibers uniquely stand as a robust, resilient and scalable solution suitable for extreme environments*

Finally, we have included the project title in the acknowledgements to exemplify high value composite structures: *"Novel fibre sensors for application in process control, evaluation and health monitoring of high-value composite assets."*

To address the comment on 'uniformity of key parameters across the fiber length/batch' being a prototype fibre, we currently limit draws to ~100's meters length scale. The fiber drawing is performed using traditional, well-established methods. Following the drip and upon reaching equilibrium in the neckdown, the draw parameters and thus the cross-section remains stable. In the manuscript on Page 6 we have provided the length achieved from one preform as 120 m, this refers to the stable length of the fibre, which have clarified. As mentioned above the OTDR did not observe any discontinuities. It is understood that larger scale draws have a longer time to reach steady state, which will only improve future uniformity. We agree that for upscaling uniformity is important, so have further elaborated by adding information on Page 6:

> *'Subsequently to the initial drip and upon reaching equilibrium in the neckdown, the draw parameters remained unchanged…. To assess uniformity, the drawn, 12 m long test piece HARFF shown in Fig. 4 (e), was cleaved 1 m intervals. The fibre showed up to ±2.3% width, ±6% thickness.'*

The reviewer questions 'robustness (mechanical stability, environmental resistance, and long-term reliability)'. In the manuscript we are clear that the HARFF is drawn from fused silica

using the same high-temperature process as conventional optical fibre, which has well-established mechanical strength, environmental stability, and long-term ageing behaviour. Further, as described in the manuscript (page 5), the fibre is coated immediately after drawing with UV-curable ORMOCER CBS-106. This coating fulfils the same role as in standard fibre, protecting the glass from surface flaws and handling-induced micro-defects, which are the primary origin of strength degradation in uncoated silica. To address this comment, we performed tensile proof testing to quantify post-draw robustness. Standard telecommunications fibres (ITU-T G.652/G.657) are proof tested at ~8.5 N. The flat fibres fabricated here withstand more than three times this load, exceeding 30 N, with testing limited by the maximum capacity of the tension system rather than fibre failure. The following clarification has been added to Page 6, Line 5 of the manuscript.

> *‘protecting the glass from surface flaws and handling-induced micro-defects, which are the primary origin of strength degradation in uncoated silica…. Six months after draw, the fibre continued to withstand proof-test loads exceeding 30 N.’*

## <u>Response to Reviewers NCOMMS-25-24356B-Z</u>

We thank the Reviewers for their careful evaluation of our manuscript and for the constructive and detailed feedback provided. We are encouraged that Reviewer #1 considers the manuscript suitable for publication and that Reviewer #3 views the work as ‘possessing an important impact on the academic communities of special fibre fabrication and fibre sensing’ and the critical perspective provided by Reviewer #4, which has helped us to more clearly distinguish the physical sensing mechanisms.

In response, we have undertaken substantial revisions to the manuscript, including additional analysis, clarification of the sensing metrics and their physical interpretation, expanded discussion of limitations and applicability, and corrections and improvements to figures, simulations, and presentation.

Below, we provide a detailed, point-by-point response to all reviewer comments and outline the corresponding changes made to the manuscript. Reviewer’s comments in **blue**; authors’ response in **black** and changes in the manuscript highlighted in blue.

### Reviewer #1

The authors have provided a comprehensive response to my questions, and I believe the revised manuscript is suitable for publication in Nature Communications.

We thank the Reviewer for this assessment. The constructive feedback provided during the review process has helped to clarify and strengthen the manuscript.

### Reviewer #3

Overall Recommendation: **Minor Revision**

1. By precisely controlling the structural parameters of the optical fiber, the author adjusted its birefringence. Can this HARFF be designed to have polarization-maintaining properties?

The measured birefringence of the HARFF designs presented ($\sim10^{-5}$) currently sits below that required for polarization maintaining operation ($10^{-4}$–$10^{-3}$), and the fibers therefore do not exhibit polarization maintaining behavior. As with circular fiber, polarization maintaining operation is feasible in principle. However, such design modifications are not pursued in this study.

**Text added, Discussion, Page 13:**

"The HARFF geometry inherently enables birefringence tuning through adjustments in aspect-ratio and loading conditions as shown in the FEM simulation. Birefringence values of the presented designs are of the order of $10^{-5}$. Further increase in birefringence sufficient to achieve polarization maintaining properties would be possible by modifying the fiber design."

2. In the case of single- and multi-core μHARFF a transmission loss of approximately 0.16 dB/m at λ = 1550 nm is estimated using cut-back technique. The loss of HARFF can be further reduced? In its fabrication process, which step is the primary factor limiting its loss? With continuous future optimization, will this fiber become comparable to conventional fiber in terms of transmission loss?

The measured propagation loss of ~0.16 dB/m reflects the current proof-of-concept implementation of the HARFF platform rather than a fundamental limitation of the geometry. At this stage, the dominant loss mechanisms are two-fold.

First, the guided cores employed here have a small semi-minor axis (~1.5–2 μm) and an elliptical cross-section, which increases confinement and bend sensitivity at 1550 nm. This is consistent with the numerical modal analysis in Fig. 5 (c). Straightforward optimisation of the core geometry, in particular increasing the minor-axis dimension, is expected to reduce this contribution.

Second, the fabrication route differs fundamentally from that used for ultra-low-loss telecommunications fibre. Commercial fibres are drawn from monolithic vapour-deposited preforms with extremely low impurity levels, whereas the present proof-of-concept HARFFs are produced using stacked XJGS1 silica wafers that include high levels of residual $OH^-$, resulting in absorption at 1550 nm. This limitation is materials-related rather than intrinsic to the HARFF geometry. With continued optimization, specifically the use of low-OH infrared-grade silica and refinement of core geometry, substantial loss reduction is expected.

**Text added, Page 12**
"The measured propagation loss of ~0.16 dB/m reflects a proof-of-concept HARFF implementation rather than a fundamental limitation of the flat-fiber geometry. Loss is primarily attributed to the small, elliptical core, which increases confinement-related bend sensitivity at 1550 nm, and to residual $OH^-$ absorption arising from the use of stacked XJGS1 silica wafers. Both effects are non-intrinsic, and further loss reduction is expected through core-geometry optimization and the use of low-OH infrared-grade silica."

3. HARFFs have enhanced mechanical properties compared to typical optical fibers for the purpose of multiparameter sensing. Therefore, the author primarily addresses the application and future development of HARFF in the field of sensing. Apart from sensing applications, can this HARFF be utilized in fields such as optical communication or laser power delivery? If feasible, what specific structural or process optimizations would be required? Conversely, if its application is challenging in these areas, what are the primary limiting factors?

As the reviewer correctly identifies, the presented HARFFs primarily target physical sensing applications. In principle, the HARFF could be adapted for optical communication upon loss reduction (see Q2) or high-power laser delivery, via utilization of either larger, multimode cores or leveraging beam delivery in a ribbon-like multi-core alignment.

> **Text added, Page 14:**
>
> “While the HARFFs demonstrated for the proof of concept are primarily designed for multiparameter sensing, the flat-fiber geometry offers potential in other specialized domains. Larger mode-area or multicore HARFF designs could enable high-power laser delivery through improved thermal coupling to a substrate, while the ribbon-like geometry may support beam-delivery or beam-combining architectures where a monolithic planar geometry is advantageous.“

4. Subfigure (b) in Fig. 4 is missing.

Figure 4 (now 3) has been revised and corrected in the revised manuscript, and the missing subfigure label (b) is now included.

5. Figure 6 demonstrates the splicing of HARFF with a conventional optical fiber. However, the author does not provide the corresponding splicing loss. The author achieved stable splicing by experimentally adjusting the splicing parameters. Given the novel structure of the fiber discussed, does achieving low-loss splicing necessitate a complex optimization process? Furthermore, for HARFF with a higher aspect ratio, does splicing with conventional single-mode fibers become significantly more challenging?

To facilitate future practical adoption within existing fiber processing techniques splicing was intentionally performed without modification to commercial splicing equipment. As stated in the original manuscript, fusion splicing was carried out using an unmodified Fujikura LZM-100 splicer. The only HARFF-specific adaptation was a simple, in-house machined flat-bottom holder that replaced the standard V-groove magnetic clamp, enabling stable mechanical support of the flat fiber geometry. No changes to splicer optics, alignment algorithms, or firmware were required.

For clarification, in **Methods** we have added a description of our HARFF-suitable splicer clamp and text added:

> **Text added to *Methods*:**
> "In comparison to the standard fiber holder, i.e., v-groove, a 1 mm wide flat-bottom slot was machined in a 2 mm diameter brass rod. A cleaved HARFF was secured in the groove using 1 mm thick rubber gasket and then the rod was placed in a standard detachable 2 mm endcap holder (Fujikura FH-100-2000). (…) The splicing parameters were fine-tuned, within original firmware boundaries, depending on HARFF geometry and splice specification as explained in Section 2."

The measured splice loss for the present HARFF design was typically < 3 dB, with variability naturally dominated by cleave quality. At present, the absence of commercially available cleavers for HARFF contributes to this variability.

For HARFF designs with higher aspect ratios, the splicing approach described here does not inherently become more problematic, provided the flexural rigidity and fiber thickness remains comparable to that of the diameter of regular circular fiber. This is because the CO□ laser in the fusion splicer interacts through a similar thickness, largely independent of changes in fiber width. Practical limitations arise primarily from the splice holder design for HARFF widths significantly exceeding ~1 mm, rather than from the splice process itself. Further reduction in splice loss is expected through tailoring HARFF core dimensions to better match the mode-field diameter of standard single-mode fibers and by minimizing modal mismatch during fusion. Importantly, these improvements do not require fundamentally new splicing techniques.

> **Text added Section 2, Page 8:**
> "The measured splice loss, typically < 3 dB, was highly dependent on HARFF cleave quality and modal-mismatch. While acceptable performance was obtained, further reduction in splice loss can be achieved by tailoring HARFF core dimensions to better match the mode field diameter of standard fibers and by minimizing modal mismatch during fusion. These straightforward optimizations, combined with precise cleaving and alignment, would enable robust integration of HARFF into existing fiber systems without requiring fundamentally new splicing techniques."
>
> **Text added to Methods:**
> "The splicing parameters were fine-tuned, within original firmware boundaries, depending on HARFF geometry and splice specification as explained in Section 2. The splice loss as estimated via HARFF transmitted power measured before and after splicing with a short (60 cm long) µHARFF section, using drops of high-index oil to reduce the impact of cladding light."

6. The Discussion section could be made more concise. Some statements have been repeated within this section and appear elsewhere in the manuscript.

The Discussion has been revised to remove repetition and improve conciseness.

7. The authors claim an improvement of "up to three orders of magnitude" in sensitivity. However, the manuscript compares a customized intensity-based metric (7.24 dB/MPa) against traditional wavelength-shift metrics. To validate this claim, please provide a calculation converting the dB variation into an equivalent refractive index change or strain resolution, allowing for a direct physical comparison with standard FBG sensors.

We agree that direct, quantitative comparison relating to refractive index-based metrics is needed.

There are two distinct performance aspects here, which are treated separately in the manuscript, these are (i) resolution based on our FP-FBG design and customized intensity-based metric and (ii) polarimetric pressure sensitivity.

In the abstract, we mention '*up to three orders of magnitude improvement in sensitivity over conventional circular cross-section optical fiber design*'. This can be shown through quantifying polarimetric pressure sensitivity. This relates to an equivalent refractive index (birefringence) change.

To extract polarimetric pressure sensitivity the FP-FBG signature response was curve fitted. Starting from a zero-birefringence assumption (maximum fringe visibility), fitting was made through the superposition of two orthogonally polarised components of equal amplitude and identical spectral envelope, representing the fast and slow axes in the fibre. Applied pressure was assumed to induce a differential wavelength separation, $\Delta\lambda$, between these components, corresponding directly to a change in phase birefringence, $\Delta n$.

The differential wavelength separation was in turn fitted to the experimentally measured FP-FBG signature using a Nelder Mead optimisation routine. This approximation yielded a polarimetric pressure sensitivity of $1.7\text{x}10^2 \pm 10\%$ rad·MPa$^{-1}$·m$^{-1}$

Conventional circular fibres with an elliptical core have reported polarimetric pressure sensitivity values, extrapolated at 1550 nm wavelength, being of the order 0.1 rad·MPa$^{-1}$·m$^{-1}$[38], hence statement of up to 3-orders of sensitivity improvement gained.

Following recommendation from the reviewer, the manuscript has been improved to include this detail. The Methods section now includes '*Birefringence and Polarimetric Pressure Sensitivity Extraction*' that explains how this measure is calculated.

**Section 2, Page 10**

"Through curve fitting experimental data (see Methods), the polarimetric pressure sensitivity was determined to be $1.7 \times 10^2 \pm 10\%$ rad·MPa$^{-1}$·m$^{-1}$. This represents

an improvement in sensitivity of up to three orders of magnitude compared with circular cross-section optical fiber containing an elliptical core [37].”

8. A hysteresis of 0.06 dB is reported. Given the peak-to-valley sensitivity, this represents a non-negligible error (approx. 3% FS). Could the authors discuss the origin of this hysteresis? Is it related to the polymeric coating or the residual stress at the CO2 laser-sealed ends?

The reported hysteresis of 0.06 dB corresponds to approximately 3% of the full-scale range, but this variation is within the noise level of the interrogator (level accuracy ±0.5 dB is considered typical for OSAs) and environmental fluctuations during calibration. We do not attribute this to polymeric coating or residual stress at the CO□ laser-sealed ends, as repeated measurements showed no systematic drift or permanent offset. Instead, the small difference likely reflects short-term thermal and mechanical stability limits of the setup rather than intrinsic fiber behavior. We clarify this point in the revised manuscript.

**Text added, Page 10:**

“Although the observed hysteresis represents ~3% FSR, it is within the noise level of the measurement system and did not indicate permanent stress effects upon testing.”

9. In Fig. 9(c), the PV response to temperature is periodic. Please clarify the monotonic dynamic range of the sensor. For the pressure sensor, does the linear fit in Fig. 8(c) represent a small section of a similar periodic Vernier function? What is the maximum measurable pressure before ambiguity occurs?

We agree that the polarimetric visibility / peak to valley (PV) response shown in Fig. 9(c) is periodic with temperature, which reflects the intrinsic 2π phase periodicity associated with birefringence-based polarimetric interrogation. However, it is important to clarify that PV is not used as the sole temperature transduction mechanism in this device. The sensor simultaneously provides two independent observables, both the PV response and the Bragg wavelength shift of the FBG, the latter exhibits a strictly monotonic and unambiguous dependence on temperature over the full measurement range.

In contrast to the temperature response shown in Fig. 9(c), the linear fit shown in Fig. 8(c) shows the initial monotonic region of the polarimetric phase response, operated below the first phase-wrapping condition. A deviation from monotonic behavior (‘roll-off’) is observed beyond approximately 4 bar, which is consistent with the onset of phase wrapping in a birefringence-based polarimetric measurement. It is important to note that the polarimetric phase does not start from zero, owing to the intrinsic birefringence of the cavity. As a result, the fundamental phase-ambiguity limit is not encountered immediately at low

pressure, and the theoretical unambiguous pressure range is significantly larger than the experimentally demonstrated range. In the present work, we intentionally restrict the measurements to the regime required to establish proof-of-concept.

More generally, the ambiguity associated with phase-periodic polarimetric sensing is not a fundamental limitation of the FP-FBG architecture. If extended dynamic range is required, established approaches such as concatenated pairs of interferometers meeting the quadrature requirement [44] can be implemented. By designing cavities with incommensurate lengths, the effective unambiguous range can be substantially increased.

We have added to the discussion of the manuscript to clarify the experimentally demonstrated range, the origin of the observed roll-off, and the straightforward routes available to extend the unambiguous operating range if required.

> **Text added, Page 12-13**
>
> “The polarimetric visibility response of the FP–FBG cavity is inherently phase-periodic due to the 2π nature of birefringence-induced phase accumulation. In the proof-of-concept study, the pressure sensor operated within the initial monotonic regime of the polarimetric response, up to approximately 0.3 MPa, beyond which phase wrapping leads to non-monotonic behavior. This operating range is sufficient for the intended demonstration and does not reflect a fundamental limitation of the architecture. If required, the unambiguous pressure range can be extended using established techniques such as multi-interferometer quadrature schemes [42].”

10. Long-term Stability of Metal-Filled Fibers: For the Sn-alloy filled sensor, did the authors perform any thermal cycling tests? Please comment on the potential delamination risks at the silica-metal interface due to the large CTE mismatch over repeated cycles.

The Sn-alloy–filled sensors were fabricated in February 2025 and were retested under identical conditions over approximately ten months, during which no measurable change in response was observed. Within the temperature range investigated, no evidence of progressive degradation was detected. The higher thermal expansion (CTE) of the alloy relative to silica is expected to generate compressive interfacial stresses during heating, which may help maintain interfacial contact.

> **Text added (Methods):**
> “The sensors were tested following approximately a 10 months ageing cycle, showing repeated characteristics. The tests involved repeated heating and cooling cycles. Within the range investigated, no evidence of progressive degradation was detected. The higher coefficient of thermal expansion of the alloy relative to silica is expected to generate compressive interfacial stresses during heating, helping to maintain interfacial contact.”

**Reviewer #4 (Remarks to the Author):**

1. In regard to the pressure sensitivity I will make a comparison to conventional single mode fibre, which has a hydrostatic pressure sensitivity of approximately 10^-11 strain/m. Value from Foster JLT, 39, 2514 (2021). This would lead to an FBG sensitivity of 16 pm/MPa. If the result in the author's paper is correct, they should have a sensitivity three orders of magnitude higher, at 16 nm/MPa. This should be easily resolvable using the author's measurement, but clearly is not. My rough estimate from the author's data is that they have a sensitivity of 8 pm/MPa, even less than standard fibre.

We agree the wording in the original manuscript was not clear. Enhancement specifically refers to pressure-induced birefringence under hydrostatic loading, rather than an enhancement of axial strain sensitivity or Bragg wavelength shift.

To avoid ambiguity, the revised manuscript now explicitly distinguishes between birefringence-based sensitivity enhancement and wavelength-shift–based sensitivity, and clarifies the physical quantity being enhanced.

> **Text added, page 2:**
> "Here, enhancement refers to an increased sensitivity linked to pressure-induced birefringence response upon hydrostatic loading, rather than an enhancement of Bragg wavelength shift associated with axial strain in conventional FBG sensors [32]."

2. The authors may benefit from performing Fourier phase analysis of their signals, which is a common approach for fibre optic interferometry.

Following the reviewer's helpful recommendation, we applied a standard procedure, now outlined in Methods, i.e., *FTT - Band-pass filter to insulate the side lobe - IFFT* of the reflected spectra for both proof-of-concept sensors in unloaded and loaded state. The unwrapped phase of the dominant Fourier component was evaluated at the central fringe (dashed in Figs. 7,8 and newly added 9) for the measurements.

The resulting phase sensitivities outlined in Results: *(i)* for the pressure sensor 31.6 rad/MPa with cross-sensitivity of 0.187 rad/K (i.e. <1%) confirming that the pressure sensor exhibits a strong pressure response while showing only a weak thermal cross-coupling, making it effectively temperature-stable for pressure measurements; *(ii)* for the temperature sensor of 6 rad/K was measured. The results have been incorporated into the revised manuscript (**Pages 10,11,13**)

> **Also, text and Fig. 9 added in Methods:**

*Fourier-Phase Analysis of FP-FBG Interferometric Signals:* To evaluate the pressure and temperature responses of the FP-FBG cavities, Fourier-phase analysis was applied to the reflected interferometric spectra. Reflected spectra were recorded using an optical spectrum analyzer (OSA). Spectra were normalized to compensate for source fluctuations. An FFT was then applied to obtain the complex spatial-frequency. The dominant peak was filtered and the phase component for the center fringe (see Fig. 7 (a,d) and Fig. 8 (b)) was unwrapped from IFFT spectra (see Fig. 9). The sensors were subjected to stimuli 0-0.2 MPa and $\Delta T_p$ 50 K, and $\Delta T_t$ 7.4 K for pressure and temperature sensor respectively, where $\Delta T_t$ represents the first sign change for ΔPV (see Fig. 8(c)). The resultant phase shifts ($\Delta\phi$) were fitted against the stimuli using a linear fit. Cross-sensitivity was determined by measuring phase change under the *non-target* measurand.

3. Fig. 1: Where does the data come from? Also, what are the two dots on the graph?

Figure 1 does not present experimental data. It shows analytically calculated values of the second moment of area about the lateral axis $I_x$ for the fibre cross sections shown, obtained directly from the standard definition of the second moment of area applied to each geometry.

As this quantity is a purely geometric property, no measurements or simulations are involved. To remove any possible ambiguity, the analytical definition of $I_x$ has now been added as Eq. (1) in the revised manuscript and the accompanying text has been clarified accordingly.

**Text added page 3**
"Fig. 1 shows the second moment of area about the lateral axis ($I_x$) for conventional optical fibre, compared to solid-core HARFF, and single channel microstructured HARFF (μHARFF), with width of 800 μm wide (see Eq. 1)."

The markers originally included in the figure were intended to indicate the specific HARFF and μHARFF geometries examined in this study. Whist revising the manuscript, we concluded that the markers did not materially improve clarity and therefore removed them from the revised Fig. 1.

4. Part 1: "I have a query relating to the numerical results in Fig. 2. The authors give Eq. 1 which relates birefringence directly to the difference in the principal components of strain in the transverse plane. To my understanding, this intrinsically assumes that the principal component directions are aligned (everywhere) to the cartesian grid. I do not believe this would be the case due to the complex geometry. The local refractive index

components would need to be evaluated considering the variation of principal strain directions across the geometry, before then calculating birefringence."

The reviewer is correct, for the general case, the principal strain directions are not necessarily aligned with a global Cartesian coordinate system. Importantly, we do not assume any such alignment in our work. We agree that showing the coordinate strains in Figure 2 (b) gives a different impression and accordingly we have changed the plots to show the principal strains. In the revised manuscript, we now make explicit that birefringence is evaluated using the local principal strains in the transverse plane of the HARFF. Our plane strain FEA model provided the local strain tensor in Cartesian coordinates as follows:

$$\varepsilon = \begin{bmatrix} \varepsilon_x & \frac{1}{2}\gamma_{xy} \\ \frac{1}{2}\gamma_{xy} & \varepsilon_y \end{bmatrix}$$

which were then transformed using the familiar 'Mohr's circle' formulation to obtain the local principal strains $\varepsilon_1$and $\varepsilon_2$:

$$\varepsilon_{1,2} = \frac{\varepsilon_x+\varepsilon_y}{2} \pm \sqrt{\left(\frac{\varepsilon_x-\varepsilon_y}{2}\right)^2 + \left(\frac{\gamma_{xy}}{2}\right)^2}$$

Noting that we have modified our notation in the revised manuscript, so that the suffices *1* and *2* denote the directions of principal strain in the fiber transverse plane.

To make this explicit, the original Eq. 1 (now Eq. 2) has been updated to clearly distinguish Cartesian strain components from principal strains. Furthermore, section '(i) Predicting birefringent response to hydrostatic pressure' now explicitly states that $\varepsilon_1$and $\varepsilon_2$ are obtained in the way described above and used directly in the birefringence calculation.

Figures 2 and 3 have been combined into Figure 2 in the revised manuscript showing the principal strain fields, derived birefringence and parametric trends within a single consistent framework, and the section '(ii) Geometric parameter sweep' updated accordingly.

4. part 2: "Further, it would be good to go a step further and calculate the birefringence of the optical modes and compare these values to the experimental results."

The primary aim of the simulations is to understand the mechanical origin of enhanced birefringence sensitivity resulting from the µHARFF geometry and to identify how cross-section geometry governs the generation of heterogeneous principal strain fields under hydrostatic loading. The finite element model was therefore intentionally kept simple to allow a systematic parameter sweep and provide clear design level insight. A fully

quantitative prediction of modal birefringence would require additional assumptions and inputs that are not presently well constrained. This includes: possible uncertainty in the strain-optic coefficients; localised residual stress arising from (a) femtosecond laser fabrication and (b) optical fibre draw history; differences between the idealised parameterised geometry used in the model and the realised fibre cross section, including dimensional tolerances and edge and corner radii. Incorporating these effects would require a substantially more complex multiphysics and fabrication informed model. Hence a qualifying statement has been added on page 4 of the revised manuscript as follows:

“It should be noted that the model is intended to be used as a comparative tool to assess the effect of varying µHARFF geometries on the *B* values at the waveguide position. To provide quantitative values of *B*, a knowledge of localised residual stress arising from the femtosecond laser fabrication of the sensor and the optical fibre draw would be required, as well as accurate representation of the geometry including dimensional tolerances and edge and corner radii.”

5. Line 157: not clear what “high confinement” means.

The term has now been explicitly defined in the manuscript. In this context, ’high confinement’ refers to strong spatial confinement of the laser beam, resulting in highly localized heating and controlled heat affected zone.

> **The following clarification has been added, Page 5:**
> “Advantages include high confinement of the laser beam and thus controlled thermal exposure, which was observed to mitigate surface-tension related deformation of the preform, associated with traditional flame-based processing methods.”

6. Line 163: more detail needed ab out the germanium-doped fused silica core rods. The diameter? Are they uniformly doped, or core clad?

Additional details have now been provided, in the Methods section of the manuscript. The fibres were fabricated using commercially supplied germanium-doped fused silica rods, which were assembled and laser-processed in-house as part of the preform fabrication process. Thorlabs Inc supplied rods used in HARFF shown in Fig. 4 (e-f) had a cladding diameter of approximately 80 µm with a ~62.5 µm Ge-doped core. Our specification corresponded to an effective core refractive index of 1.47, however we were informed that the specific radial dopant profile of the commercial rods is proprietary to the manufacturer and is therefore not known in detail.

> **Text added in Methods:**
> “(…)the fibers were drawn using preforms that were assembled and laser-processed in-house. To obtain a preform, fused silica wafers (XJGS1) and

germanium-doped fused silica rods (GIF625, Thorlabs Inc) with the cladding diameter etched to approximately 80 µm and the core of 62.5 µm ($n_{core}$ ≈ 1.4749, at λ = 1300 nm),”

7. Line167: what laser wavelength is used? I see it’s in the methods section. I believe directly referencing to the methods section could be used in the main text as some information is provided in the main text, but not all, making it a bit confusing.

The Fabrication section has been revised to explicitly state the laser wavelength used, to improve clarity and avoid the need to cross-reference the Methods section.

**The revised text now reads (Page 5):**

“First, 1 mm thick flat silica-wafers and a germanium-doped fused silica core rods ($n_{core}$ ≈ 1.4749, at $\lambda$ = 1300 nm) were laser-cut to the desired dimensions, using a focused $CO_2$ laser (λ=10.6 µm). Next, the preform stack was assembled and laser-welded along the edges to create permanent and robust bonds between each component in the stack. This selective welding allowed the definition of an air-channel geometry in the HARFF, essential in the manufacturing process of µHARFF. To create the joint between the components in the stack, a defocused $CO_2$ laser spot size of approximately 2 mm and a laser power of up to 60 W was used.”

8. Fig. 4: It’s not quite clear what the image with “weld” is showing. Is this the edge of the two-channel fibre?

The image shows a cross-section through the weld obtained in the typical µHARFF preform, now encircled in the revised Fig. 4. To clarify this, we revised Fig. 4 (b) to improve its clarity.

**Figure 4 caption now reads:**

“(…) micrograph (b) shows a typical cross-section of the weld with visible chipping in the diced-through microstructure (…)”

9. Fig. 5c: the colour bar tick labels are quite small and the image seems to be low resolution.

The heat map in **Fig. 5 (c)** has been updated in the revised manuscript with increased resolution and enlarged colour bar tick labels.

10. Splice losses should be mentioned with respect to Fig. 6.

Splice losses are now explicitly evaluated and discussed in relation to Fig. 6, with the measurement method described in the Methods section.

**Text added Section 2, Page 8**:

The measured splice loss, typically < 3 dB was highly dependent on HARFF cleave quality and modal-mismatch. While in this work, acceptable performance was obtained, further reduction in splice loss can be achieved by tailoring HARFF core dimensions to better match the mode field diameter of standard fibers and by minimizing modal mismatch during fusion. These optimizations, combined with precise cleaving and alignment, would enable robust integration of HARFF into existing fiber systems without requiring fundamentally new splicing techniques.

**Text added in Methods***:*

The splice loss as estimated via HARFF transmitted power measured before and after splicing with a short (60 cm long) μHARFF section, using drops of high-index oil to strip cladding mode light from measurement.

11. The referencing to Fig. 6c and Fig. 6b seems to be incorrect in lines 221 and 223.

The figure references in lines 221 and 223 have been corrected in the revised manuscript.

12. Line 269: I believe this should be 10^-2 dB/K.

The text has been revised to state 8×10^−3 dB/K, for clarity.

13. Fig. 8c: Why are there only errors bars for the depressurizing results? What are the error bars representing?

The error bars in Fig. 8(c) represent the pressure resolution of the inline manometer used during the measurements. In the original version of the figure, error bars were shown only for the depressurising cycle for visual clarity. We agree that this presentation should be improved, so Fig. 8(c) has therefore been revised to include identical error bars for both the pressurising and depressurising cycles.

14. Fig. 9a: Why does the fibre appear to be tapered?

The apparent tapering visible in the rendered image arises from the chosen perspective in the three-dimensional illustration rather than from a physical variation in fibre geometry. We have clarified in the revised caption for Figure 9.

**Figure 9 caption, modified:**

Fig. 9 Sketch (a) of the alloy filled sensor based on a single channel HARFF spliced with circular fiber (with consistent cross-section along the length shown in Fig. 6 (c)); the inset shows its micrograph, bottom illumination used; (b) spectral response of the center fringe in the sensor, and (c) its sensitivity demonstrated up to 12 pm/K and ±0.075 dB/K in 20 K periods.

15. Fig. 9 (c): colour code the y-axes to the data, maybe also add arrows so that it's clear which axes the two sets of data correspond to.

The y-axes in Fig. 9(c) have now been colour-coded to match the corresponding data sets, and arrows have been added to clearly indicate the association between each data set and its respective axis.

16. Line 382: coreless fibre is mentioned but I can't see where this was used in the main text (ignore if I just missed this).

Coreless fibre was used during splicing to coreless HARFF structures as the coreless section facilitated subsequent waveguide writing, as described in the Methods section. To remove ambiguity, the role of the coreless HARFF is now explicitly stated in the revised manuscript.

**Text added to the Methods section, page 13:**
"In the case of coreless HARFF (e.g., Fig. 4 (c,d)) a short section of coreless fiber (FG125LA, Thorlabs Inc) was used as an intermediate splice to accommodate small alignment tolerances between SMF-28 core and thin-wall of μHARFF. Subsequently, a rectangular cross-section waveguide(…)"

17. There are many grammar errors throughout the paper, some examples:
a. Unnecessary commas on line 26
b. Lines 26-29, sentence is too long
c. Unnecessary comma on line 40
d. Missing comma after waveguide on line 45
e. Line 65, "Fibres" should be "Fibre"
f. Capitalisation is used when acronyms are defined, which is unneceesary (except

proper nouns)
g. Line 81, “HARFF pressure sensor can achieve sensitivity a resolution of …” needs to be corrected
h. Line 156, “The HARFF preforms utilize a custom”, odd language to say that the preform “utilizes” something
i. Line 279, “As a sensing element…”
j. Line 317: “This was made possible…”

The manuscript has been thoroughly revised for clarity, grammar, and consistency. All of the specific issues identified by the reviewer, including punctuation, sentence structure, terminology, and phrasing, have been corrected. In addition, the manuscript has undergone a comprehensive language edit to ensure consistent usage, clear definition of acronyms, and improved readability throughout.

# Response to Reviewers NCOMMS-25-24356C

We thank the Editor and the Reviewers for their careful evaluation of our manuscript and for the constructive and detailed feedback provided. We are encouraged that Reviewer #1 and Reviewer #3 considers the manuscript suitable for publication. The critical perspective provided by Reviewer #4, has helped us to further clarify the manuscript for broad readership of Nature Communications.

In response, we have added further clarification of physical interpretation of the results.

Below, we provide a detailed, point-by-point response to R#3 comments and outline the corresponding changes made to the manuscript. Reviewer's comments in **blue**; authors' response in **black** and changes in the manuscript highlighted in blue.

## Reviewer #1

The authors have provided a comprehensive response to my questions, and I believe the revised manuscript is suitable for publication in Nature Communications.

## Reviewer #3

The author has addressed those specific concerns. I believe the manuscript, in its current form, is suitable for publication in the Nature Communications

We sincerely thank both Reviewers for their careful evaluation of our manuscript and for their thoughtful and constructive feedback throughout the review process. We are grateful for their positive assessment of the revised version and are pleased that they consider the manuscript suitable for publication in Nature Communications.

Their comments have been instrumental in strengthening the clarity, rigor, and overall presentation of the work.

## Reviewer #4 (Remarks to the Author):

The authors have satisfactorily addressed the technical aspects of my previous review.

We thank Reviewer #4 for their careful reassessment of the manuscript and for confirming that the technical aspects raised in the previous review have been satisfactorily addressed.

I still believe that the pressure sensitivity results are inadequate to justify publication in Nature Communications. The results in Fig. 7c are based on intensity changes rather than a wavelength shift, which is due to the wavelength shift being quite small.

First, we respectfully clarify that the mechanism in Fig. 7 (c) is **not based on wavelength shift sensing**. The device is a Fabry Perot Bragg grating (FP FBG) structure in which the pressure response is mediated primarily through stress induced changes in cavity birefringence, which in turn modify the cavity finesse and therefore the fringe contrast (visibility). The reported observation is consequently a contrast modulation within the interference packet, rather than a Bragg peak displacement. In this architecture, the Bragg wavelength shift is intentionally minimal. The *near zero* pressure-driven wavelength shift was thus expected and consistent with the design.

We have revised the manuscript to make this distinction explicit, clearly separating finesse modulation-based interrogation from conventional wavelength shift based FBG sensing, and stating why the absence of a large wavelength shift is intrinsic to this device operation.

> **Text added Methods, Page 15:**
>
> Here, the pressure response arises from a stress-induced birefringence modification within the cavity, which changes the cavity finesse and therefore the fringe contrast. The observed signal is thus an intensity modulation associated with a change in interference pattern visibility.

Second, we emphasise that the pressure experiment is provided as a proof-of-principle demonstration of stress optic birefringence engineering within our novel microstructured high aspect ratio flat fibre platform, rather than as the sole or primary claim. The central contribution of our work is the new fibre geometry, fabrication route, and sensor concept, which enables a planar, compact, and minimally intrusive platform that supports device architectures and integration scenarios that are not readily accessible using conventional circular fibres and external mechanical transducers. We have strengthened the discussion, (see page 12) to further emphasis the purpose of the pressure sensing demonstration, ensuring that the context is clear.

This indicates that the intrinsic sensitivity is low and the observed intensity changes could quite easily come from other factors affecting light transmission.

We respectfully disagree with the implication that the reported signal is susceptible to generic transmission fluctuations, because the metric used is **ratiometric**.

Specifically, the FP FBG response is extracted using a **fringe contrast (peak to valley (PV))** metric within the same interference packet (equivalently, fringe visibility), rather than using an absolute optical power level. Any multiplicative transmission variations that affect both peak and trough similarly (for example, source power drift, coupling changes, connector loss) cancel in the ratio, rendering the measurement largely immune to common mode transmission perturbations. The measured contrast modulation therefore reflects a pressure induced

modification of cavity birefringence and finesse, rather than a global change in transmitted intensity.

To avoid ambiguity, we have added explicit wording in the manuscript describing the ratiometric extraction and its immunity to common mode transmission changes.

**Text added, Page 10:**

*It is important to note that the PV power level is insensitive to common mode drift, because it is evaluated within the same fringe packet.*

In any case, the error bars indicate a sensing resolution the order of 0.1 MPa, which only has limited applications.

The error bars in Fig. 7c do not represent the sensing resolution of the FP-FBG readout. They correspond to the pressure axis of the plot (i.e. horizontal) and therefore indicate the accuracy and discretization of the applied pressure measurement from the manometer, which is +/-0.02 MPa and not 0.1 MPa. Therefore, the error bars reflect uncertainty in the calibrated pressure axis and not the uncertainty in the optical measurement, plotted on the vertical axis of Fig. 7c. To make matters absolutely clear we have added a note in the caption of Figure 7.

The intrinsic sensor response extracted from the FP-FBG fringe contrast yields a sensitivity of 7.24 dB/MPa. The achievable pressure resolution is therefore set by the dynamic range and noise floor of the optical interrogation and the PV contrast extraction, rather than by the manometer step size used in the calibration. To avoid ambiguity, we have revised the manuscript to explicitly distinguish calibration uncertainty from optical resolution and to state that, under standard optical interrogation, the demonstrated intrinsic sensitivity corresponds to kilopascal level resolution.

**Text added, Page 2:**

For instance, leveraging 80 μm thick and 655 μm wide microstructured HARFF as pressure sensor, inherent sensitivity resolution in order of single kilopascal was demonstrated using standard optical interrogation, thereby representing an improvement of up to three orders of magnitude over the earlier mentioned similar sensors constructed in commercial silica fibers.
